# Trends in Selenium Utilization in Marine Microbial World Revealed through the Analysis of the Global Ocean Sampling (GOS) Project

**DOI:** 10.1371/journal.pgen.1000095

**Published:** 2008-06-13

**Authors:** Yan Zhang, Vadim N. Gladyshev

**Affiliations:** Department of Biochemistry, University of Nebraska, Lincoln, Nebraska, United States of America; Progentech, United States of America

## Abstract

Selenium is an important trace element that occurs in proteins in the form of selenocysteine (Sec) and in tRNAs in the form of selenouridine. Recent large-scale metagenomics projects provide an opportunity for understanding global trends in trace element utilization. Herein, we characterized the selenoproteome of the microbial marine community derived from the Global Ocean Sampling (GOS) expedition. More than 3,600 selenoprotein gene sequences belonging to 58 protein families were detected, including sequences representing 7 newly identified selenoprotein families, such as homologs of ferredoxin–thioredoxin reductase and serine protease. In addition, a new eukaryotic selenoprotein family, thiol reductase GILT, was identified. Most GOS selenoprotein families originated from Cys-containing thiol oxidoreductases. In both Pacific and Atlantic microbial communities, SelW-like and SelD were the most widespread selenoproteins. Geographic location had little influence on Sec utilization as measured by selenoprotein variety and the number of selenoprotein genes detected; however, both higher temperature and marine (as opposed to freshwater and other aquatic) environment were associated with increased use of this amino acid. Selenoproteins were also detected with preference for either environment. We identified novel fusion forms of several selenoproteins that highlight redox activities of these proteins. Almost half of Cys-containing SelDs were fused with NADH dehydrogenase, whereas such SelD forms were rare in terrestrial organisms. The selenouridine utilization trait was also analyzed and showed an independent evolutionary relationship with Sec utilization. Overall, our study provides insights into global trends in microbial selenium utilization in marine environments.

## Author Summary

Selenium (Se) is an essential micronutrient due to its requirement for biosynthesis and function of the 21^st^ amino acid, selenocysteine (Sec). Sec is found in the active sites of selenoproteins, most of which exhibit redox function, in all three domains of life. In recent years, genome sequencing projects provided a large volume of nucleotide and protein sequence information. Identification of complete sets of selenoproteins (selenoproteomes) of individual organisms and environmental samples is important for better understanding of Se utilization, biological functions of this element, and changes in Se use during evolution. Here, we describe a comprehensive analysis of the selenoproteome of the microbial marine community derived from the Global Ocean Sampling (GOS) expedition. More than 3,600 selenoprotein gene sequences belonging to 58 protein families were detected and analyzed. Our study generated the largest selenoproteome reported to date and provided important insights into microbial Se utilization and its evolutionary trends in marine environments.

## Introduction

Selenium (Se) is an essential trace element that exerts a number of health benefits yet is required only in small amounts [1]–[3]. It is incorporated into selenoproteins, many of which are important antioxidant enzymes, in all three domains of life, and occurs in these proteins in the form of selenocysteine (Sec), the twenty-first amino acid in the genetic code [4]–[6]. Sec insertion is specified by a UGA codon, which is normally read as a stop signal. The decoding of UGA as Sec requires a translational recoding process that reprograms in-frame UGA codons to serve as Sec codons [5]–[8]. The mechanisms of selenoprotein biosynthesis have been the subject of numerous studies [5], [7]–[12]. The translation of selenoprotein mRNAs requires both a *cis*-acting selenocysteine insertion sequence (SECIS) element, which is a hairpin structure residing in 3′-untranslated regions (3′-UTRs) of selenoprotein mRNAs in eukaryota and archaea, or immediately downstream of Sec-encoding UGA codons in bacteria [7], [13]–[16], and several *trans*-acting factors dedicated to Sec incorporation [7],[17].

In recent years, an increase in the number of genome sequencing projects combined with the rapidly emerging area of microbial metagenomics provided vast amount of gene and protein sequence data. However, selenoprotein genes are almost universally misannotated in these datasets because of the dual function of UGA codon. To address this problem, a variety of bioinformatics approaches have been developed and used for selenoprotein searches in both prokaryotes and eukaryotes [18]–[24]. With these programs, researchers successfully identified complete sets of selenoproteins (selenoproteomes) of individual organisms and environmental samples [20]–[26].

In early 2007, three papers from the J. Craig Venter Institute were published reporting the results of the first phase of the large-scale metagenomic sequencing project – Global Ocean Sampling (GOS) expedition, a comprehensive survey of bacterial, archaeal and viral diversity of the world's oceans [27]–[29]. The general objective of this project was to expand our understanding of the microbial world by studying the gene complement of marine microbial communities. A metagenomics approach was used to sequence DNA isolated from selected sites of the aquatic microbial world. The previous Sargasso Sea project [30], which reported environmental shotgun sequencing of marine microbes in the nutrient-limited Sargasso Sea, was considered as a pilot study for the subsequent GOS project. The GOS dataset encompasses 44 sequenced samples from diverse aquatic (largely marine) locations which contain a total of ∼7.7 million sequencing reads and more than 8 billion nucleotides [29]. These data not only provide opportunities for the identification and characterization of genes, species and communities, but have potentially far-reaching implications for biological energy production, bioremediation, and creating solutions for reduction/management of greenhouse gas levels.

Within this framework, identification and characterization of selenoproteins in such a huge metagenomic dataset can shed light on the roles of Se in marine microbial communities. Previously, we examined the microbial selenoproteome of the Sargasso Sea via searches for Sec/cysteine (Cys) pairs in homologous sequences [25]. This method performed well and further research has shown that it is reliable in identifying selenoproteins in both organism-specific and environmental genomes [24],[26],[31].

In this study, we utilized a similar approach to analyze the distribution and composition of marine selenoproteins in the GOS shotgun dataset. More than 3,600 selenoprotein genes were detected, which is ten times the number of selenoproteins in the Sargasso Sea study. Several novel prokaryotic selenoprotein families were predicted. We further analyzed the dataset in various ways deriving insights into global trends in Se utilization.

## Results

### General Features of the GOS Selenoproteome

**Figure 1:**
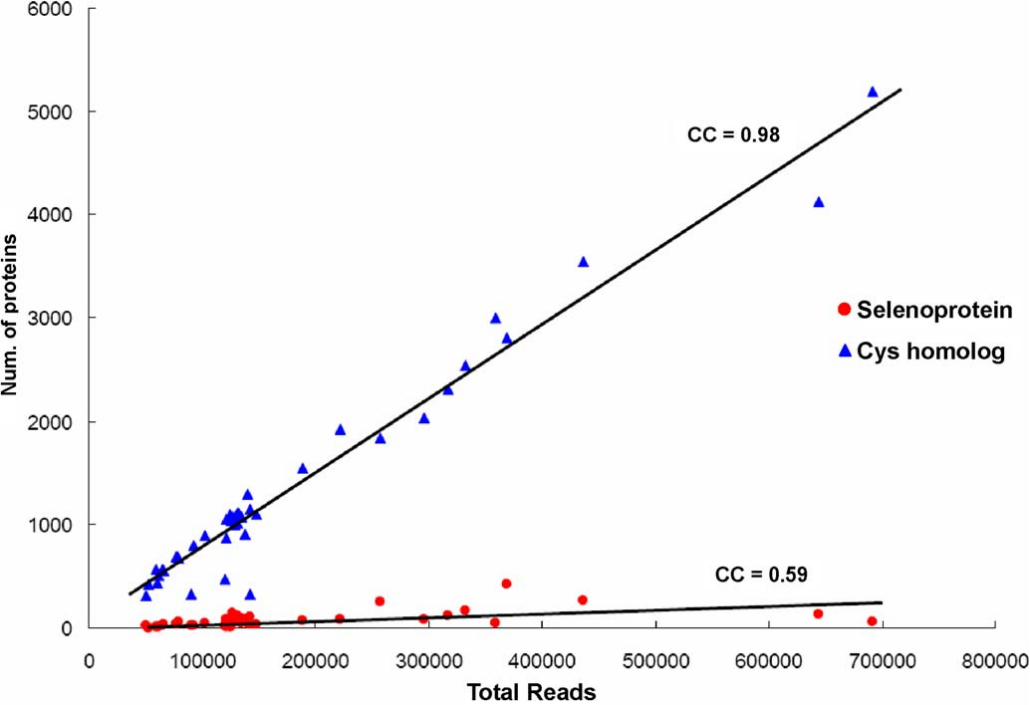
Distribution of selenoproteins and Cys-containing homologs in GOS samples. Each point shows the number of selenoproteins or Cys-containing homologs detected relative to sample size (i.e., total number of sequenced reads) for each GOS sample. Correlation coefficients (CCs) are shown for selenoproteins and their Cys homologs. The number of Cys-containing homologs of selenoproteins correlated with sample size, whereas selenoproteins showed a poor correlation.

**Figure 2:**
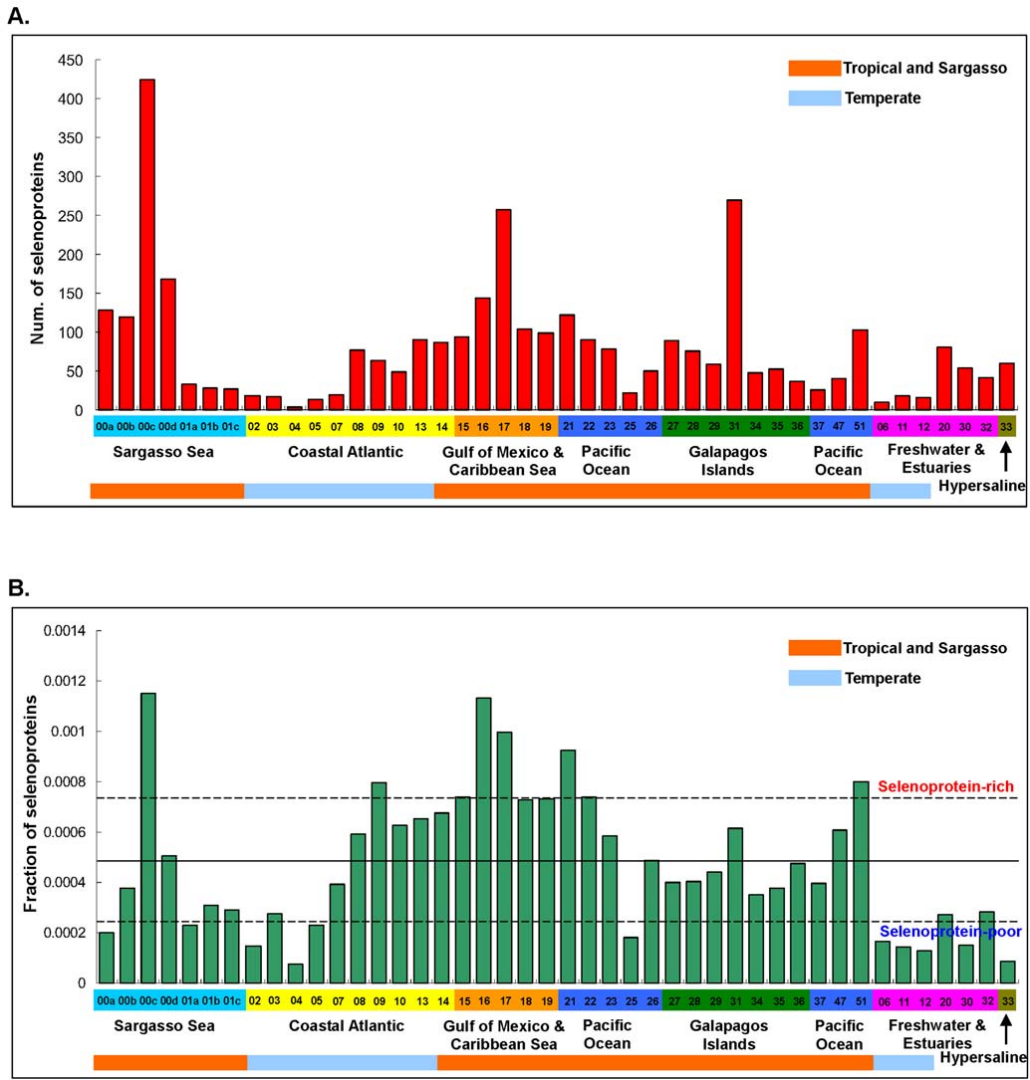
Composition of selenoprotein population along the GOS transect. Color scheme is used to show different environments. A. Number of selenoproteins in individual samples; B. Normalized occurrence of selenoproteins. Boundary lines for selenoprotein-rich and -poor samples are shown. Samples 01a, 01b and 01c were obtained using different size filters at the same collection site as described in the text.

**Figure 3:**
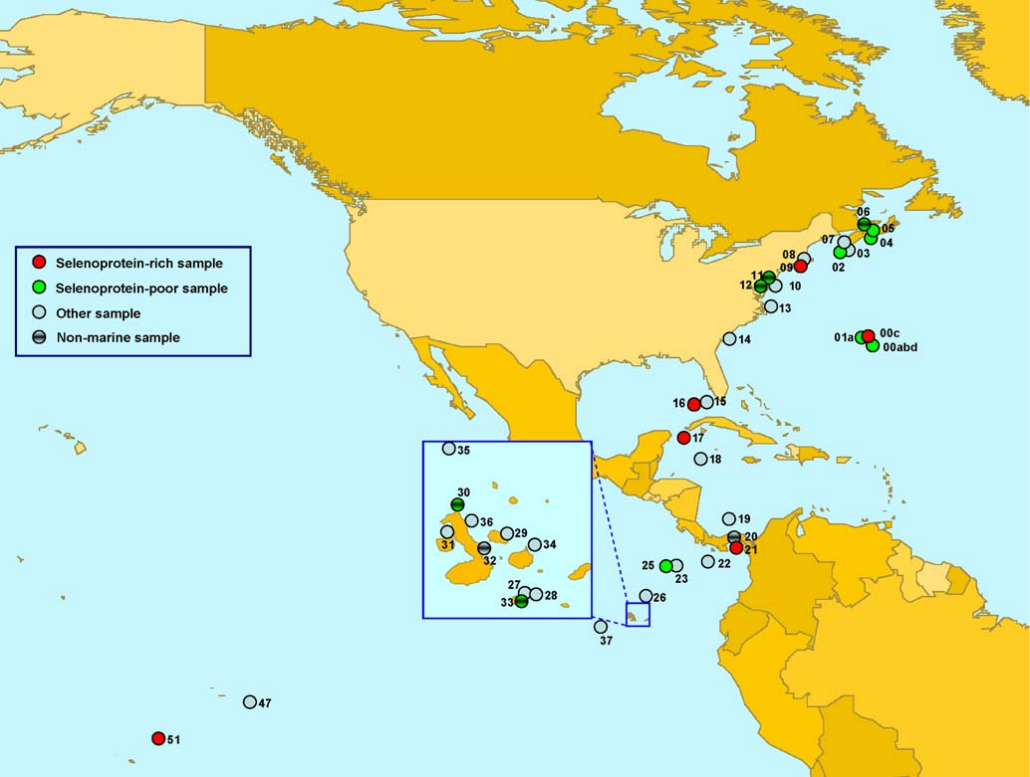
Geographic locations of GOS samples. Planiglobe tool (http://www.planiglobe.com/omc_set.html) was used to generate the map. 44 samples (excluding 13 partially sequenced samples) are shown in the map, along the eastern North American coast through the Gulf of Mexico into the equatorial Pacific. Selenoprotein-rich and selenoprotein-poor samples are represented in red and green, respectively. Locations of seven nonmarine aquatic samples are also shown.

Computational analysis of 44 sequenced GOS samples identified 3,506 selenoprotein sequences that belonged to previously described selenoprotein families (Table 1, all sequences are available in supplemental Dataset S1). We also identified 58,225 Cys-containing homologs of these selenoproteins in the GOS sequences. Canonical correlation analysis of their occurrence based on sample size (i.e., total number of sequenced reads for each sample) showed a strong correlation between the number of Cys-containing homologs and sample size (correlation coefficient, CC, is 0.98), but selenoproteins showed a weak correlation (CC is 0.59), suggesting widely different utilization of Sec in GOS samples (Figure 1). The samples were then clustered in various ways based on geographic location, water temperature (tropical or temperate), and salinity (sea water, fresh water, estuaries, or hypersaline lake). GS00c (Sargasso Sea Station 3, 425 selenoproteins, 12.1% of all detected selenoproteins), GS31 (coastal upwelling near Galapagos Islands, 269 selenoproteins, 7.7%) and GS17 (Yucatan Channel in Caribbean Sea, 257 selenoproteins, 7.3%) had the highest numbers of selenoproteins (Figure 2A). Normalized occurrence of selenoproteins is shown in Figure 2B (on average, GOS samples had 0.047% reads containing selenoprotein genes). We designated samples as selenoprotein-rich (6 samples) if they contained 1.5 times the average level and selenoprotein-poor (11 samples) if they had twice less the average level of selenoproteins (Figure 2B). Geographically selenoprotein-rich and -poor samples did not cluster with each other, arguing against significant geographic differences in Sec utilization within the areas examined by the GOS project (Figure 3). It should be noted that except for the Sargasso Sea samples (GS00a–GS01c), all other samples were collected in daytime between August 2003 and May 2004, but most of them were collected in a narrow time period (November 2003∼March 2004, see Table 1 for sample date and time). Therefore, seasonal and yearly shifts in microbial community were considered to be small. However, it would be of interest to examine the contribution of seasonal factors to changes in the detected microbial selenoproteome once sufficient sampling becomes available.

**Table 1:** Distribution of known selenoproteins and Cys-containing homologs in GOS samples.

Sample ID	Habitat type	Date (mm/dd/yy)	Time	Temperature (°C)	Total reads	Selenoprotein families	Num. of selenoproteins	Num. of Cys homologs
***Marine samples (Open ocean and coastal)***								
**GS00a	Open ocean	02/26/03	3:00 & 10:00	20.0	644,551	20	128	4,129
GS00b	Open ocean	02/26/03	3:35 & 10:43	20.0	317,180	23	120	2,316
*GS00c	Open ocean	02/25/03	13:00	19.8	368,835	36	425	2,805
GS00d	Open ocean	02/25/03	17:00	20.0	332,240	25	168	2,544
**GS01a	Open ocean	05/15/03	11:40	22.9	142,352	13	33	322
GS01b	Open ocean	05/15/03	11:40	22.9	90,905	14	28	331
GS01c	Open ocean	05/15/03	11:40	22.9	92,351	12	27	804
**GS02	Coastal	08/21/03	6:32	18.2	121,590	9	18	872
GS03	Coastal	08/21/03	11:50	11.7	61,605	8	17	509
**GS04	Coastal	08/22/03	5:25	17.3	52,959	2	4	419
**GS05	Embayment	08/22/03	16:21	15	61,131	5	14	439
GS07	Coastal	08/25/03	8:25	17.9	50,980	11	20	318
GS08	Coastal	11/16/03	16:45	9.4	129,655	16	77	1,075
*GS09	Coastal	11/17/03	10:30	11	79,303	15	63	680
GS10	Coastal	11/18/03	4:30	12	78,304	15	49	694
GS13	Coastal	12/19/03	6:28	9.3	138,033	21	90	907
GS14	Coastal	12/20/03	17:12	18.6	128,885	18	87	1,012
GS15	Coastal	01/08/04	6:25	25	127,362	19	94	1,078
*GS16	Coastal Sea	01/08/04	14:15	26.4	127,122	24	144	1,048
*GS17	Open Ocean	01/09/04	13:47	27	257,581	29	257	1,843
GS18	Open Ocean	01/10/04	8:12	27.4	142,743	22	104	1,146
GS19	Coastal	01/12/04	9:03	27.7	135,325	20	99	1,076
*GS21	Coastal	01/19/04	16:48	27.6	131,798	19	122	1,012
GS22	Open Ocean	01/20/04	16:39	29.3	121,662	15	90	1,047
GS23	Open Ocean	01/21/04	15:00	28.7	133,051	17	78	1,115
**GS25	Fringing Reef	01/28/04	10:51	28.3	120,671	13	22	469
GS26	Open Ocean	02/01/04	16:16	27.8	102,708	11	50	890
GS27	Coastal	02/04/04	11:41	25.5	222,080	21	89	1,920
GS28	Coastal	02/04/04	15:47	25.0	189,052	16	76	1,549
GS29	Coastal	02/08/04	18:03	26.2	131,529	9	58	1,108
GS31	Coastal upwelling	02/10/04	14:43	18.6	436,401	22	269	3,544
GS34	Coastal	02/19/04	17:06	27.5	134,347	12	47	1,072
GS35	Coastal	03/01/04	16:44	21.8	140,814	13	53	1,290
GS36	Coastal	03/02/04	12:52	25.8	77,538	11	37	694
GS37	Open Ocean	03/17/04	16:38	28	65,670	9	26	563
GS47	Open Ocean	03/28/04	15:25	28.6	66,023	14	40	556
*GS51	Coral Reef Atoll	05/22/04	7:04	27.3	128,982	16	103	1,003
***Nonmarine samples***								
**GS06	Estuary	08/23/03	10:47	11.2	59,679	6	10	570
**GS11	Estuary	11/18/03	11:30	11	124,435	11	18	1,096
**GS12	Estuary	12/18/03	11:32	1	126,162	9	16	1,035
GS20	Fresh Water	01/15/04	10:24	28.6	296,355	14	80	2,034
**GS30	Warm Seep	02/09/04	11:42	26.9	359,152	11	54	3,002
GS32	Mangrove	02/11/04	11:30	25.4	148,018	15	42	1,102
**GS33	Hypersaline	02/19/04	13:35	37.6	692,255	19	60	5,187
**Total**					**7,689,374**		**3,506**	**58,225**

*: Selenoprotein-rich sample

**: Selenoprotein-poor sample

It has been reported that GOS samples grouped based on sequence similarity and taxonomy correlate with environmental parameters of GOS sites, particularly with regard to water temperature and salinity [29]. We found that except for sample GS09, all selenoprotein-rich samples belonged to the marine “tropical & Sargasso” group which had an average sampling temperature at 25.5°C. Also, all samples from the Gulf of Mexico and Caribbean Sea (GS15–GS19) showed elevated levels of selenoproteins (Figure 2B), suggesting an active utilization of Sec in this area. In contrast, more than half of selenoprotein-poor samples (6 out of 11) were derived from temperate water area (12.1°C in average). This observation is consistent with our previous hypothesis that the use of Sec increases at higher temperature [32]. Besides, 5 of 7 nonmarine aquatic samples were selenoprotein-poor and the remaining two were borderline selenoprotein-poor (Figure 2B). These nonmarine samples were geographically distant (Figure 3) and located in different temperature zones. Further analysis of these samples with regard to habitat and environment suggested one likely factor, salinity, which was different between marine (including both open ocean and coastal areas) and nonmarine environments. Except for GS33 which was sampled from a hypersaline lagoon (salinity is 63.4 ppt) and showed low species richness [29],[33], all nonmarine aquatic samples were characterized by very low salinity (<4 ppt) [29]. This observation suggested that fresh water or low-salinity aquatic environments may work against Sec utilization. Although more extensive sample classification was difficult because of their number, water depth, fresh water input, proximity to land and filter size all appeared to affect Sec abundance to some extent. For example, the filter for most samples was 0.1∼0.8 µm, which concentrated mostly bacterial and archaeal microbial populations [29]. However, among the Sargasso Sea samples, GS01a, GS01b and GS01c were three subsamples from the same site, representing three distinct size fractions (3.0–20, 0.8–3.0, and 0.1–0.8 µm, respectively). This feature explains the fact that GS01a was relatively poor in selenoproteins even though it was located in the area rich in selenoproteins. Similarly, GS25, another selenoprotein-poor sample, was collected using a larger filter (0.8–3.0 µm). No conclusion could be made regarding the relationship between nutrients (such as carbon, nitrogen and phosphorus) and Sec utilization. For example, in the nutrient-limited Sargasso Sea, both selenoprotein-rich (GS00c) and selenoprotein-poor (GS00a) samples were found. Similar observations were observed for coastal waters and estuaries where nutrients are more abundant, and for the open ocean where nutrients are limited. Additional factors, such as organism density, ecosystem complexity, light for phototrophs and fixed carbon/energy for chemotrophs may ultimately affect Sec utilization in microbial communities and warrant further studies once additional sequences become available.

Selenoproteins detected through the homology-based procedure (see details in Materials and Methods) belonged to 51 previously described selenoprotein families (Table 2, details are shown in Table S1). Most of these families had much more Cys-containing homologs than selenoproteins in the GOS dataset. All selenoprotein families previously detected in the Sargasso Sea were identified in the current GOS dataset, including prominent selenoproteins: SelW-like, selenophosphate synthetase (SelD), proline reductase PrdB subunit, peroxiredoxin (Prx), thioredoxin (Trx), glutaredoxin (Grx) and a variety of Prx-like/Trx-like/Grx-like proteins [25]. Other selenoproteins included a UGSC-containing protein (one of the major selenoprotein families in GOS samples, U is a one letter designation for Sec) and several selenoproteins identified in various metagenomic sequencing projects [26],[31]. In addition, we identified a large number of distant homologs of Prx-like/Trx-like selenoproteins. In order to analyze them against previously identified Prx-like/Trx-like proteins, we clustered these proteins into different subfamilies based on conserved domain classification (Pfam/COG), motif features and phylogenetic analysis. Several selenoproteins were represented by single sequences only, e.g., glycine reductase selenoprotein A (GrdA) and heterodisulfide reductase subunit A (HdrA). In this case, sequencing errors that generated in-frame TGA codons could not be excluded; however, the fact that they corresponded to known selenoproteins and also possessed strong SECIS elements strongly suggested that they were true selenoproteins. 20 selenoprotein families were represented by more than 40 selenoprotein sequences and accounted for more than 94% of all selenoprotein sequences. Similar to the selenoproteome of the Sargasso Sea, the most abundant selenoprotein families were SelW-like, SelD, UGSC-containing protein, Prx, PrdB, and different subfamilies of Prx-like/Trx-like/Grx-like proteins. The current version of GOS selenoproteome has become the largest selenoproteome identified to date, and its analysis greatly expands our understanding of Sec utilization in microbial marine communities.

**Table 2:** Summary of 51 prokaryotic selenoprotein families identified in GOS samples.

Prokaryotic selenoprotein family	Domains (Pfam/COG/CDD)	Sec-containing motif	Num. of selenoproteins	Num. of samples containing selenoprotein	Num. of Cys homologs in GOS samples
SelW-like*	pfam05169	CxxU	427	40	56
SelD	cd02195	-	357	40	558
Trx-like 1*	COG0526	UxxC	321	42	7,880
AhpD-like 1*	COG2128	CxxU	299	37	902
UGSC-containing*	-	UxxC	255	37	21
Prx*	pfam00578	TxxU	253	34	7,348
Proline reductase PrdB*	pfam07355	UxxC	235	38	110
Prx-like 1*	pfam00578	UxxC	225	37	2,740
Trx-like 3*	cd02950 (e-value>0.001)	UxxC	212	33	183
Prx-like 2*	pfam04592 (e-value>0.001)	UxxC	99	23	100
FdhA	COG0243	-	95	15	2,271
Arsenate reductase (ArsC) 1*	pfam03960	UxxS	77	23	784
COG0737, UshA-like	COG0737	CxU	76	20	92
AhpD-like 2*	pfam02627	CxxU	70	25	207
Prx-like 3*	pfam08534 (e-value>0.001)	UxxC	68	20	2,608
DsbA 1*	pfam01323	UxxC	63	23	704
Prx-like (UGC-containing)*	pfam00578	UGC	46	15	855
Grx-like*	pfam00462 (e-value>0.001)	UxxC	42	15	255
Arsenate reductase 2*	cd03032	UxxC	42	21	238
Glutathione peroxidase (GPx)*	COG0386	-	40	18	3,287
Deiodinase-like	pfam00837	-	22	12	21
Trx-like 2 (Thiol∶disulfide interchange protein)*	cd02953	UxxC	22	13	2,098
MoeB	pfam05237	-	19	7	2,262
DsrE 2*	pfam02635	UxxC	19	8	15
DsbA 2*	pfam01323 (e-value>0.001)	UxxC	18	10	235
Unknown	-	-	15	9	0
Glutathione S-transferase (GST)	COG0625	-	12	8	1,036
Methionine sulfoxide reductase A (MsrA)*	pfam01625	-	9	7	4,678
Rhodanase-related sulfurtransferase*	cd01449	-	8	6	3,367
Arsenite S-adenosylmethyltransferase	PRK11873	-	8	4	245
Sulfurtransferase COG0607*	COG0607	-	6	4	4,277
GrdB*	pfam07355	UxxC	5	3	75
OsmC-like protein*	pfam02566	UxxT	5	3	161
DsbA 3*	pfam01323	UxxV	5	3	72
DsbG-like*	COG1651	UxxC	5	5	728
NADH∶ubiquinone oxidoreductase subunit E	pfam02508	-	3	3	1,484
FrhD*	pfam02662	CxxU	3	2	4
OS_HP2	pfam07610	UxC	2	2	354
Mercuric transport protein MerP	-	-	2	1	0
Putative mercuric transport protein	-	-	2	1	1
Sulfurtransferase homologous to a rhodanese-like protein*	COG0607 (e-value>0.01)	-	2	2	19
Grx*	pfam00462	UxxC	2	2	1,952
DsrE 1*	-	UxxC	2	2	0
OS_HP1	-	UxxC	1	1	69
GrdA	pfam04723	CxxU	1	1	0
Cation-transporting ATPase, E1-E2 family	pfam00122	UxxC	1	1	882
Methylated-DNA-protein-cysteine methyltransferase	pfam01035	-	1	1	2,879
NADH oxidase	PRK09564	-	1	1	99
Sulfurtransferase COG2897*	COG2897	-	1	1	8
YHS domain protein (OS)	-	-	1	1	0
Heterodisulfide reductase, subunit A*	pfam00070	CxxU	1	1	5
**Total**			**3,506**		**58,225**

*: Homologs of known thiol-based oxidoreductases or Trx-like fold proteins

**Figure 4:**
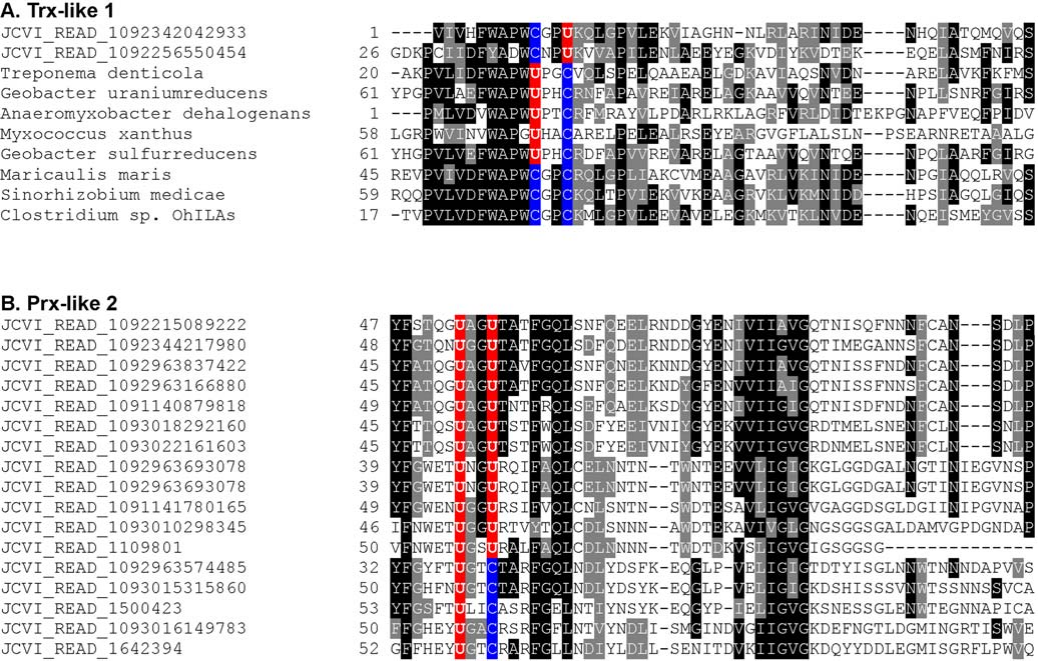
Multiple sequence alignments of known selenoprotein families containing new Sec positions. The alignments show Sec-flanking regions in detected selenoproteins. Conserved residues are highlighted. Sec (U) and the corresponding Cys (C) residues are shown in red and blue, respectively. A. Trx-like 1 family; B. Prx-like 2 family.

Most selenoproteins with known function are oxidoreductases, and among 51 selenoprotein families detected in GOS samples, 33 (2887 sequences, 82.3%) were homologs of known thiol oxidoreductases or possessed Trx-like fold (Table 2). Many of these selenoproteins contained a conserved UxxC/UxxS/CxxU/TxxU redox motif. In a small number of known selenoprotein genes, new Sec positions were identified. For example, a new redox motif (CxxU) was detected in Trx-like 1 family (COG0526, TrxA, thiol-disulfide isomerase and thioredoxins) which normally contains a UxxC motif (i.e., in all previously identified sequences) (Figure 4A). In addition, several UxxU-containing sequences were detected in a Prx-like 2 family (low similarity to pfam04592, Selenoprotein P N-terminal region), which is a very distant homolog of known Prxs and has no strong homolog in any of the sequenced prokaryotic genomes (Figure 4B).

**Figure 5:**
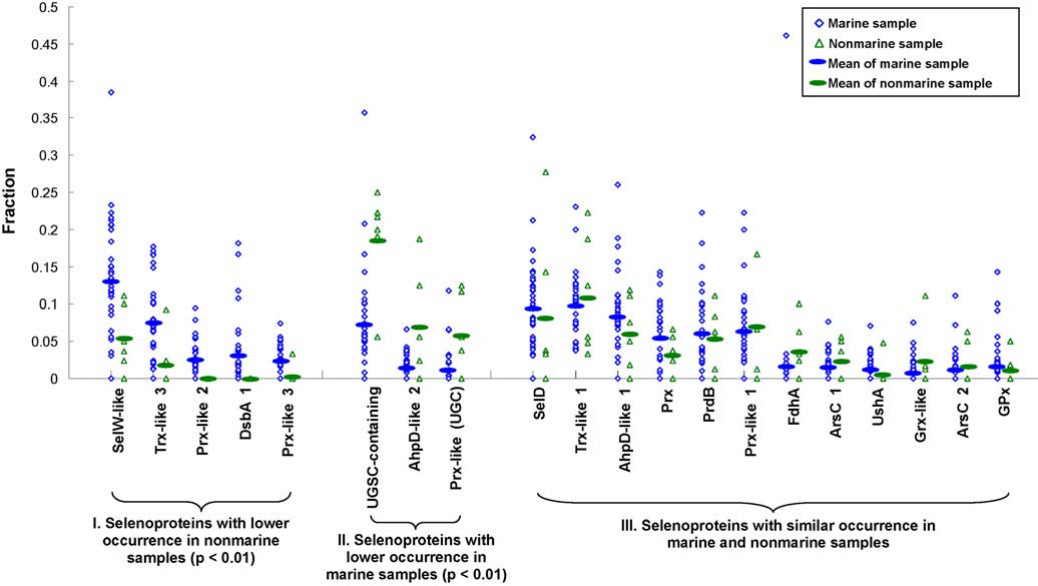
Distribution of selenoprotein families in different habitat types. Occurrence (as fraction of all proteins in a sample) of 20 most abundant selenoprotein families in samples from different environments is shown. Mean values of occurrence of each family in different habitats are also indicated. Selenoprotein families were clustered into three groups: (I) selenoproteins with lower occurrence in nonmarine samples (i.e., average occurrence in marine samples is more than twice that in nonmarine samples), (II) selenoproteins with lower occurrence in marine samples (average occurrence in nonmarine samples is more than twice that in marine samples), and (III) selenoproteins with similar occurrence in marine and nonmarine samples. T-test was used to assess statistical difference among marine and nonmarine habitats for indicated selenoprotein families.

To further investigate the relationship between occurrence of selenoprotein families and sample features (e.g., marine versus nonmarine), we analyzed the most abundant selenoprotein families in each GOS sample separately (Table 3). Excluding the samples containing a small number of selenoproteins (≤15), the majority selenoprotein families showed a similar occurrence in marine and nonmarine aquatic samples. In contrast, several selenoprotein families appeared to be differentially distributed. For example, SelW-like protein was generally the most abundant selenoprotein family in marine samples, whereas the UGSC-containing protein was most frequently utilized in nonmarine samples. As discussed above, salinity appears to be a factor that influences (perhaps indirectly) selenoprotein utilization. Figure 5 shows the occurrence of 20 most abundant selenoprotein families based on habitat. T-test was used to assess occurrence of each of these families in marine and nonmarine habitats. This analysis showed that occurrence of selenoprotein families in group I (selenoproteins with lower occurrence in nonmarine samples, Figure 5) and II (selenoproteins with lower occurrence in marine samples) were statistically different between marine and nonmarine samples (p<0.01).

**Table 3:** Top three selenoproteins in each GOS sample.

Sample ID	Selenoprotein families	Most abundant selenoprotein families and percentage of total		
		No. 1	No. 2	No. 3
***Marine samples (Open ocean and coastal)***				
**GS00a	20	FdhA (46.1%)	SelW (10.9%)	SelD (7.8%)
GS00b	23	SelW (14.2%)	PrdB (11.7%)	Prx-like 1 (10.8%)
*GS00c	36	Prx (12.7%)	SelW (11.5%)	SelD (9.6%)
GS00d	25	Trx-like 1 (12.5%)	SelW (11.3%)	PrdB (10.1%)
**GS01a	13	PrdB (18.2%)	Prx-like 1 (15.2%)	Dio-like, Trx-like 1 (12.1%)
GS01b	14	GPx, Prx (14.3%)	Prx-like 1, DsbA 1 (10.7%)	Dio-like, Trx-like 1 (7.1%)
GS01c	12	SelW (22.2%)	Trx-like 3 (14.8%)	Prx-like 1, AhpD-like 1 (11.1%)
**GS02	9	PrdB (22.2%)	UGSC-containing, DsbA 1 (16.7%)	Trx-like 1, ArsC 2 (11.1%)
GS03	8	Trx-like 3, AhpD-like 1 (17.6%)	SelW, SelD, Prx-like (UGC), Trx-like 1, DsbA 1 (11.8%)	Prx-like 1 (5.9%)
**GS04	2	UGSC-containing (75%)	PrdB (25%)	-
**GS05	5	UGSC-containing, PrdB (35.7%)	Prx (14.3%)	Prx-like 1, ArsC 1 (7.1%)
GS07	11	Prx-like 1, Trx-like 1 (20%)	PrdB, MsrA, GPx (10%)	SelD, UGSC-containing, MoeB, Prx-like 3, Trx-like 2 (5%)
GS08	16	UGSC-containing (20.8%)	AhpD-like 1 (15.6%)	PrdB, Prx-like 1 (9.1%)
*GS09	15	UGSC-containing, Trx-like 1 (14.3%)	PrdB (12.7%)	SelD, AhpD-like 1 (11.1%)
GS10	15	SelW (18.4%)	UGSC-containing, Trx-like 1, Trx-like 3, AhpD-like 1 (10.2%)	SelD, Prx, Prx-like 1 (8.2%)
GS13	21	Prx-like 1 (22.2%)	PrdB, Prx, Trx-like 1 (7.8%)	Prx-like (UGC), AhpD-like 2 (6.7%)
GS14	18	SelW (18.4%)	PrdB (12.6%)	Prx, Trx-like 1, AhpD-like 1 (10.3%)
GS15	19	SelW (16.0%)	Prx (13.8%)	UGSC-containing (10.6%)
*GS16	24	SelD (12.5%)	Prx (10.4%)	SelW (9.7%)
*GS17	29	SelD (12.5%)	PrdB (9.8%)	Prx (9.0%)
GS18	22	SelW, SelD (14.4%)	UGSC-containing (11.5%)	Trx-like 1 (8.7%)
GS19	20	SelW (13.1%)	Prx, AhpD-like 1 (10.1%)	PrdB (9.1%)
*GS21	19	SelD (13.1%)	SelW (12.3%)	Trx-like 1 (10.7%)
GS22	15	SelW, Trx-like 1 (20%)	Trx-like 3 (16.7%)	SelD (12.2%)
GS23	17	SelW (20.5%)	SelD (14.1%)	Trx-like 1 (12.8%)
**GS25	13	DsbA 1 (18.2%)	Trx-like 1 (13.6%)	SelW, Dio-like, MsrA, Prx (9.1%)
GS26	11	AhpD-like 1 (26%)	Trx-like 1 (20%)	SelW (14%)
GS27	21	SelD (15.7%)	SelW (13.5%)	Trx-like 1, AhpD-like 1 (11.2%)
GS28	16	Trx-like 3 (17.1%)	SelW (11.8%)	SelD, Trx-like 1 (10.5%)
GS29	9	SelW (20.7%)	SelD (17.2%)	Trx-like 3 (15.5%)
GS31	22	AhpD-like 1 (14.5%)	SelD (11.9%)	Trx-like 1 (11.2%)
GS34	12	SelW, SelD (21.3%)	Trx-like 3 (14.9%)	PrdB, AhpD-like 1 (8.5%)
GS35	13	AhpD-like 1 (18.9%)	SelW (15.1%)	SelD (13.2%)
GS36	11	SelD (32.4%)	SelW (21.6%)	Trx-like 1, Trx-like 3 (10.8%)
GS37	9	SelW (38.5%)	Trx-like 1 (23.1%)	Prx, ArsC 1, AhpD-like 1 (7.7%)
GS47	14	SelW, PrdB (15%)	SelD, UGSC-containing, Trx-like 1, Trx-like 3, AhpD-like 1 (10%)	Prx-like 1 (5%)
*GS51	16	SelW (23.3%)	Trx-like 3 (16.5%)	SelD (13.6%)
***Nonmarine samples***				
**GS06	6	AhpD-like 1 (40%)	Trx-like 1 (20%)	SelW, SelD, PrdB, Trx-like 3 (10%)
**GS11	11	UGSC-containing (22.2%)	SelW, PrdB, Grx-like, AhpD-like 1 (11.1%)	Prx, Prx-like (UGC), Trx-like 1, Trx-like 2, ArsC 1 (5.6%)
**GS12	9	UGSC-containing (25%)	AhpD-like 2 (18.8%)	Prx-like (UGC), Trx-like 1 (12.5%)
GS20	14	UGSC-containing (20%)	Trx-like 1 (18.8%)	AhpD-like 2 (12.5%)
**GS30	11	SelD (27.8%)	Trx-like 1 (22.2%)	Prx-like 1 (16.7%)
GS32	15	UGSC-containing (19.0%)	Prx-like 1 (16.7%)	SelD (14.3%)
**GS33	19	UGSC-containing (21.7%)	Prx-like (UGC) (11.7%)	PrdB (8.3%)

*: Selenoprotein-rich sample

**: Selenoprotein-poor sample

### Identification of New Selenoproteins in GOS Samples

**Figure 6:**
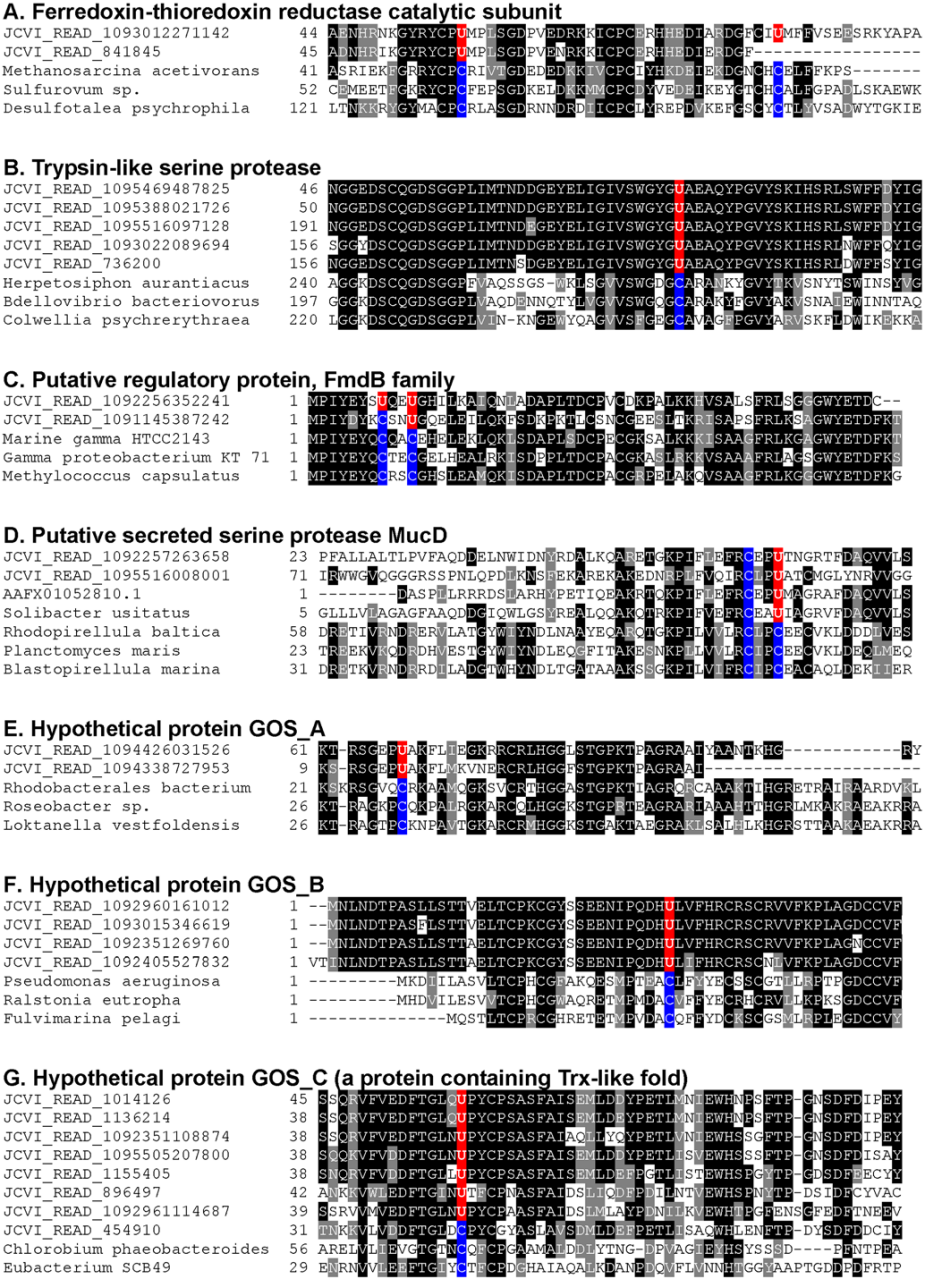
Multiple sequence alignments of new selenoproteins and their Cys homologs. New selenoprotein sequences detected in the GOS database and their Sec/Cys-containing homologs present in indicated organisms are shown. Predicted Sec (U) and the corresponding Cys (C) residues in other homologs are shown in red and blue background, respectively. A. Ferredoxin-thioredoxin reductase catalytic subunit; B. Trypsin-like serine protease; C. Putative regulatory protein, FmdB family; D. Putative secreted serine protease MucD; E. Hypothetical protein GOS_A; F. Hypothetical protein GOS_B; G. Hypothetical protein GOS_C (a protein containing Trx-like fold).

Besides known selenoproteins, we identified 7 new selenoprotein families (Table 4, all sequences are available in supplemental Dataset S2). They were represented by 2–11 individual TGA-containing sequences except for a hypothetical protein GOS_C which had 74 selenoprotein sequences. Among 7 new families, four either contained a domain of known function or were homologous to protein families with known/predicted functions. Particularly interesting was identification of ferredoxin-thioredoxin reductase (FTR) catalytic subunit and trypsin-like serine protease homologs. FTR is a key enzyme of the ferredoxin/thioredoxin system, which catalyzes reduction of thioredoxins with light-generated electrons [34]–[36]. Two Cys residues constitute a redox-active disulfide bridge functional in the reduction of Trx [37]. We identified two FTR selenoprotein sequences, including one (JCVI_READ_1093012271142) which contained two predicted Sec residues exactly corresponding to the two redox-active Cys residues (Figure 6A). Location of these Secs indicates functionality of these residues.

**Table 4:** Novel selenoproteins identified in the GOS database.

Protein family	Number of selenoproteins	Number of Cys homologs	Conserved domains
Ferredoxin-thioredoxin reductase	2	10	COG4802, Ferredoxin-thioredoxin reductase catalytic subunit
Trypsin-like serine protease	9	128	COG5640, Secreted trypsin-like serine protease
Putative regulatory protein, FmdB family	2	807	COG2331, Uncharacterized protein conserved in bacteria
Putative secreted serine protease MucD	2	12	-
Hypothetical protein GOS_A	2	155	-
Hypothetical protein GOS_B	11	125	-
Hypothetical protein GOS_C	74	312	-
**Total**	**102**	**1,549**	

**Figure 7:**
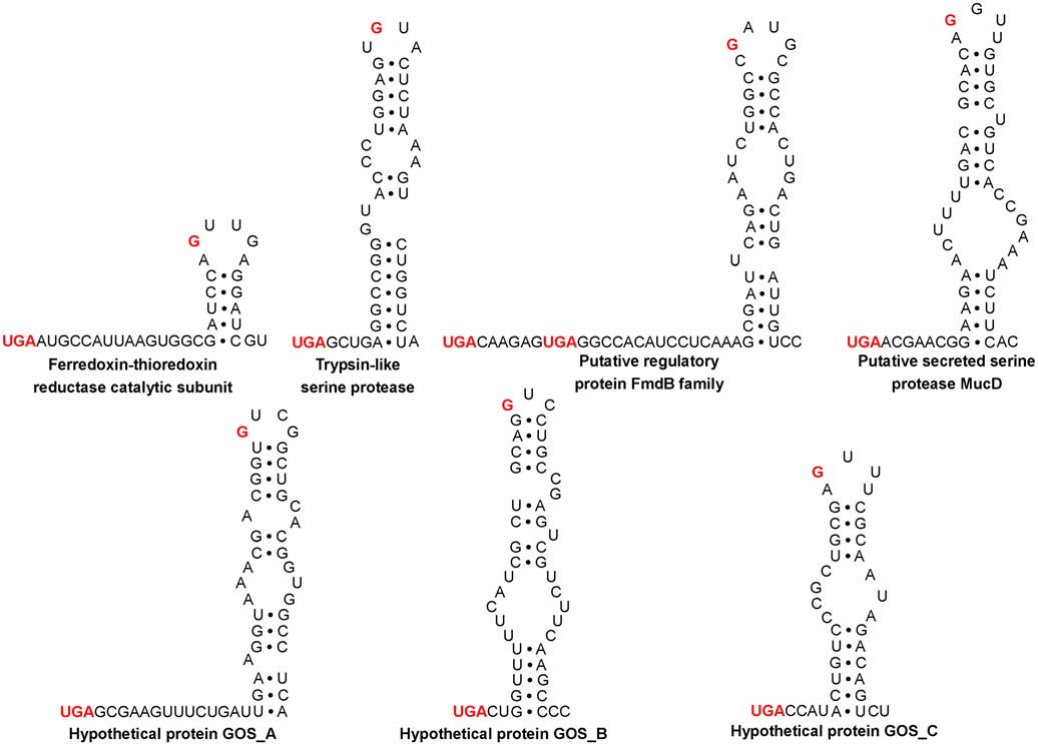
Predicted bacterial SECIS elements in representative sequences of new selenoprotein families. Only sequences downstream of in-frame UGA codons are shown. In-frame UGA codons and conserved guanosines in the apical loop are shown in red. Ferredoxin-thioredoxin reductase, JCVI_READ_1093012271142; Trypsin-like serine protease, JCVI_READ_733735; Putative regulatory protein FmdB family, JCVI_READ_1092256352241; Putative secreted serine protease MucD, JCVI_READ_1092257263658; Hypothetical protein GOS_A, JCVI_READ_1094426031526; Hypothetical protein GOS_B, JCVI_READ_1092960161012; Hypothetical protein GOS_C, JCVI_READ_1092961114687.

Trypsin is a well-known serine protease which catalyzes the hydrolysis of peptide bonds. No redox function has been reported for members of this family. We found 9 selenoprotein sequences containing the trypsin-like domain (COG5640, secreted trypsin-like serine protease) and the predicted Sec corresponded to a conserved Cys residue within this domain, suggesting a potential redox function for this Cys (Figure 6B). No functional evidence could be obtained for hypothetical proteins GOS_A∼GOS_C. However, a Trx-like fold and a conserved UxxC motif were present in GOS_C, suggesting that this protein may have a thiol-based redox function. Multiple alignments of these new selenoproteins and their Cys-containing homologs (Figure 6A–6G) highlight sequence conservation of Sec/Cys pairs and their flanking regions. New selenoproteins contained stable bacterial SECIS-like elements downstream of Sec-encoding TGA codons (Figure 7). In addition, we detected several TGA-containing sequences, which showed similarity neither to known and new selenoproteins nor to each other. Some of them contained candidate SECIS elements. However, no definitive conclusions could be made regarding these sequences because of the possibility of sequencing errors. Future experimental verification is needed for these selenoprotein candidates.

### Distinguishing Marine Prokaryotic and Eukaryotic Selenoproteins

Previous analyses revealed that several selenoprotein families occur in both prokaryotes and eukaryotes, e.g., SelW-like, GPx and deiodinase [25]. Recently, additional such selenoprotein families were identified, e.g., methionine sulfoxide reductase A (MsrA), Prx, SelL (a Prx-like protein), arsenite S-adenosylmethyltransferase (PRK11873, arsM) and several Prx-like/Trx-like proteins [31], [38]–[40]. Most eukaryotic species containing these selenoproteins are aquatic organisms (such as green algae and fish). In the GOS sequence dataset, more than 90% sequences are derived from bacteria whereas only 2.8% could be definitively assigned to the eukaryotic domain [27]. To distinguish bacterial and eukaryotic selenoproteins, we employed several approaches including phylogenetic analyses and investigation of eukaryotic SECIS elements. Our results suggested that all detected new and known selenoproteins that occur in both prokaryotes and eukaryotes could be assigned to the bacterial domain.

In addition, several eukaryotic selenoproteins were detected in different GOS samples by homology analysis using known eukaryotic selenoproteins, including protein disulfide isomerase (PDI), SelM, SelT, SelU and thioredoxin reductase (data not shown). Although most of the reads containing these selenoprotein genes were too short to investigate the presence of eukaryotic SECIS element in 3′-UTR, phylogenetic analyses and the absence of bacterial SECIS elements suggested that these sequences are eukaryotic.

**Figure 8:**
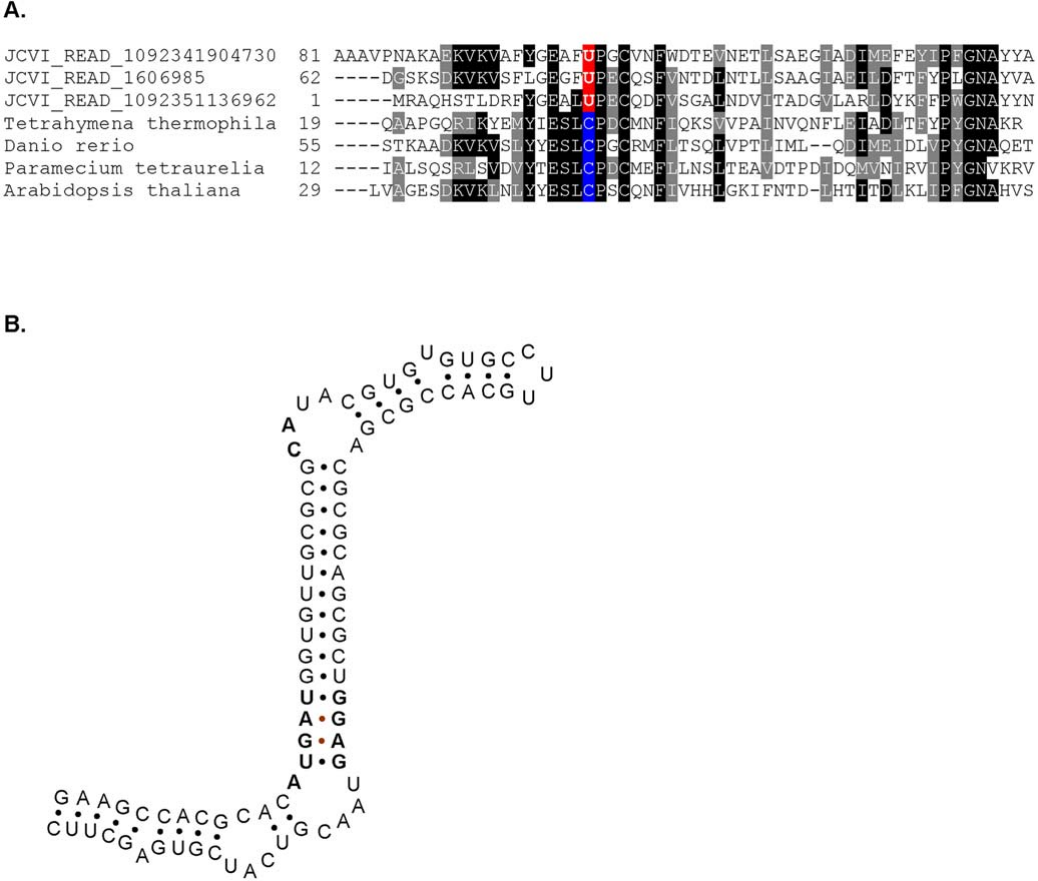
A new eukaryotic selenoprotein GILT. A. Multiple alignment of selenoproteins detected in the GOS database and their Cys-containing homologs present in indicated organisms; B. Predicted eukaryotic SECIS element (JCVI_READ_1092351136962).

Interestingly, a new eukaryotic selenoprotein family, gamma-interferon-inducible lysosomal thiol reductase (GILT), was also detected. GILT is a key enzyme to facilitate complete unfolding of proteins destined for lysosomal degradation by releasing structural constraints imposed by intra- and inter-chain disulfide bonds [41],[42]. No homologs of this protein are known in prokaryotes. In this study, we identified three selenoprotein sequences for this family. A eukaryotic SECIS element predicted by SECISearch [18] was found in the 3′-UTR of one selenoprotein gene, providing additional evidence that they are eukaryotic GILT selenoproteins. Multiple alignment of GILT sequences and the predicted eukaryotic SECIS element are shown in Figure 8.

### Novel Domain Fusions Involving Selenoproteins

**Figure 9:**
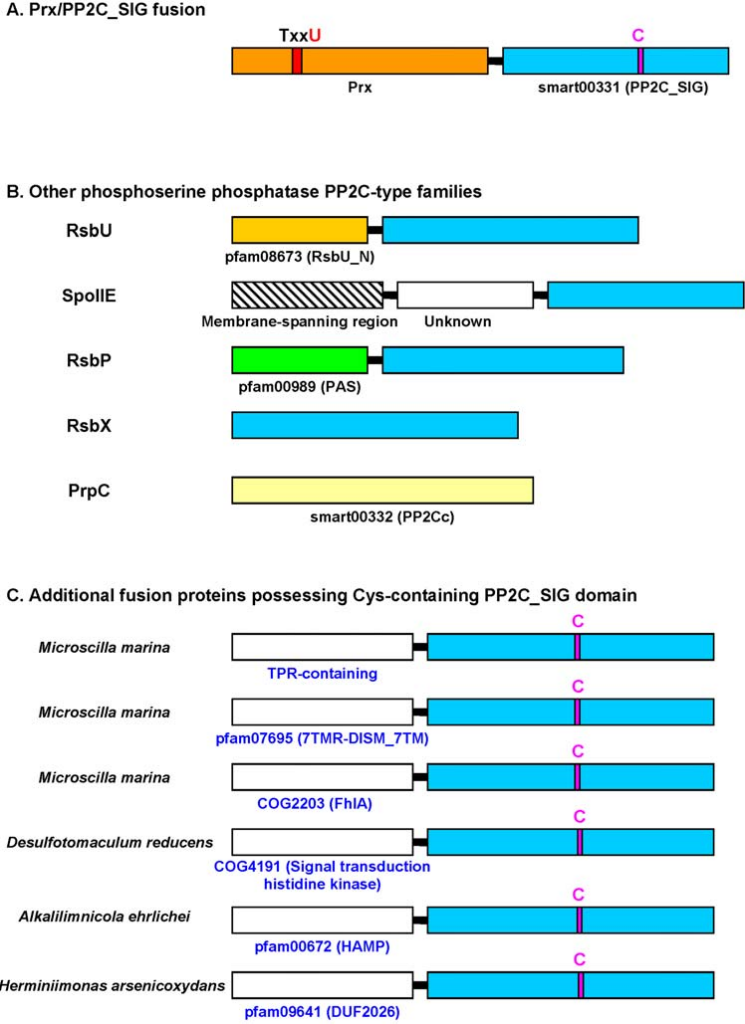
Domain organization of different PP2C-type phosphatase families. A. Prx-fused PP2C-type phosphatase family which contains a conserved Cys; B. Other phosphoserine phosphatase PP2C-type families; C. Additional fusion proteins possessing Cys-containing PP2C-type phosphatase domain. Location of conserved Cys is shown.

**Figure 10:**
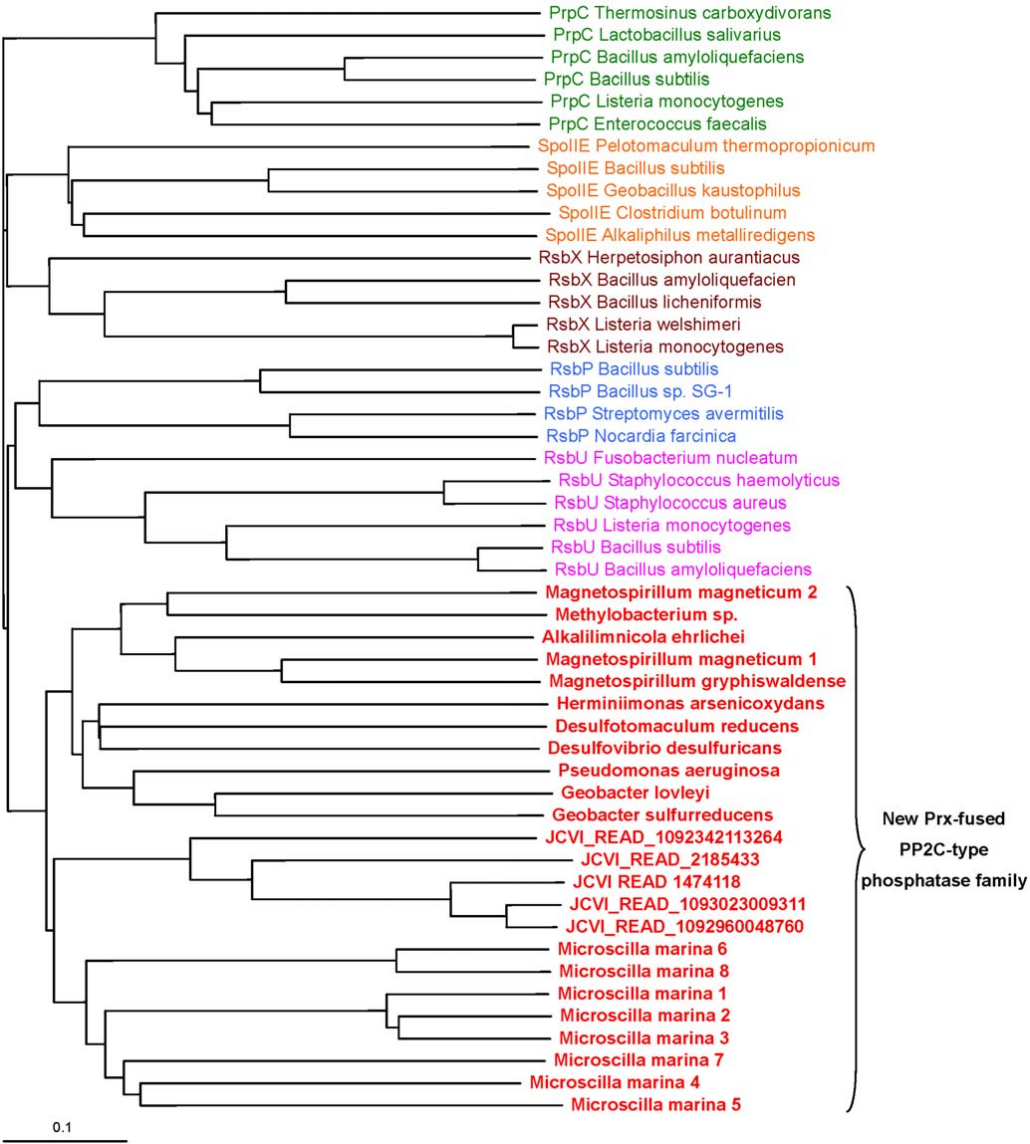
Phylogenetic tree of different PP2C-type phosphatase families. The new Prx-fused PP2C-type phosphatase proteins are shown in red and bold. Note that almost all sequenced organisms containing this family are shown (particularly in *M. marina* where more than 150 members of this family could be detected). Other PP2C-type phosphatase families are shown in different colors. Measurement of distance for the branch lengths (shown by a bar) is indicated.

**Figure 11:**
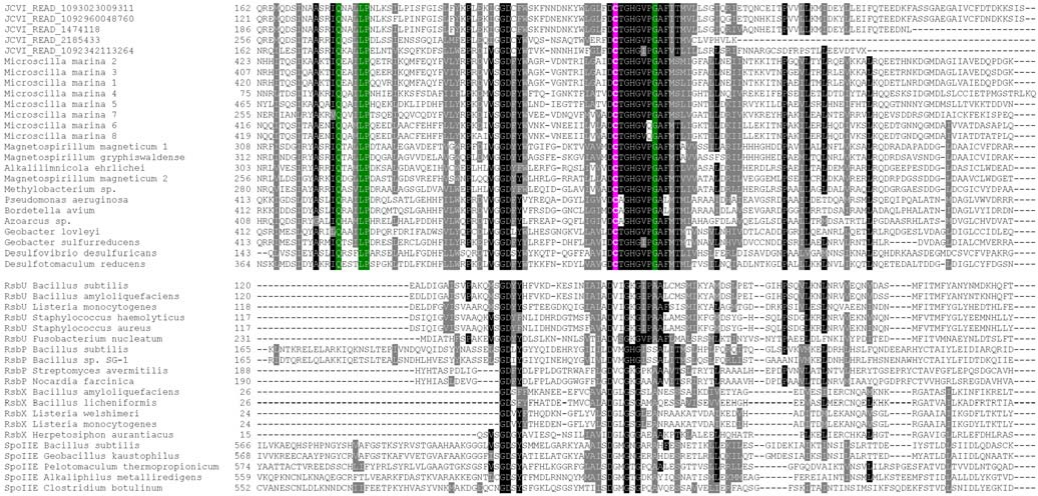
Multiple alignment of different PP2C-type phosphatase families. The alignment shows the phosphatase regions in detected proteins. Conserved residues are highlighted. The conserved Cys in the new PP2C-type phosphatase family is shown in pink background. Other residues which are present in this new family but absent in other families are shown in green background.

We identified novel domain fusions in several selenoprotein families. One example involved Prx that was fused with a distant homolog of PP2C-type phosphatase (smart00331, PP2C_SIG, Figure 9A). The PP2C-type phosphatase superfamily includes several subgroups, such as RsbU that contains an additional N-terminal domain (pfam08673, RsbU_N) and acts as a positive regulator of the activity of σ^B^, the general stress-response σ factor of gram positive microorganisms [43],[44]. Other PP2C-type phosphatase subfamilies include PrpC, SpoIIE, RsbP and RsbX [45]–[49], in which the PP2C-type phosphatase domains are fused with different domains (Figure 9B). We further checked the occurrence of this distant PP2C-type phosphatase in all sequenced bacteria and found orthologs only in a limited number (no more than 20) of organisms in different bacterial phyla and fused with different domains (Figure 9C). Phylogenetic analyses suggested that the Prx-fused phosphatases form a separate group within the PP2C-type phosphatase superfamily (Figure 10). Multiple alignments showed that several conserved residues are specific for this subgroup, especially a Cys residue which is present in all members of the Prx-fused subgroup but absent in other PP2C-type phosphatase subfamilies (Figure 11). This conserved Cys may also have a redox function. Surprisingly, one marine gliding bacterium, *Microscilla marina*, the only organism containing the Prx-fused phosphatase domain in Bacteroidetes, possessed a large number of such proteins. Compared to other organisms which contained only 1–2 members, 159 individual sequences containing this phosphatase subfamily were identified in *M. marina*, all of which had the conserved Cys residue and were fused with different domains, suggesting a particular importance of this distant PP2C-type phosphatase subfamily in this marine organism.

Additional examples of domain fusions are shown in Figure S1. Functions of most of these domains are not clear. However, as a rule, at least one conserved Cys was present in these sequences, suggesting a potential redox activity. For example, the UGSC-containing protein which likely has a Trx-like fold was fused with a conserved domain (designated Unknown_1, Figure S1A). Unknown_1 protein was also present in a limited number of aquatic organisms. Another example involved the fusion of a Prx-like 3 and Unknown_3 domain (Figure S1D). There were three conserved Cys residues in Unknown_3, including a conserved CxxC motif which may have a thiol-based redox function.

**Figure 12:**
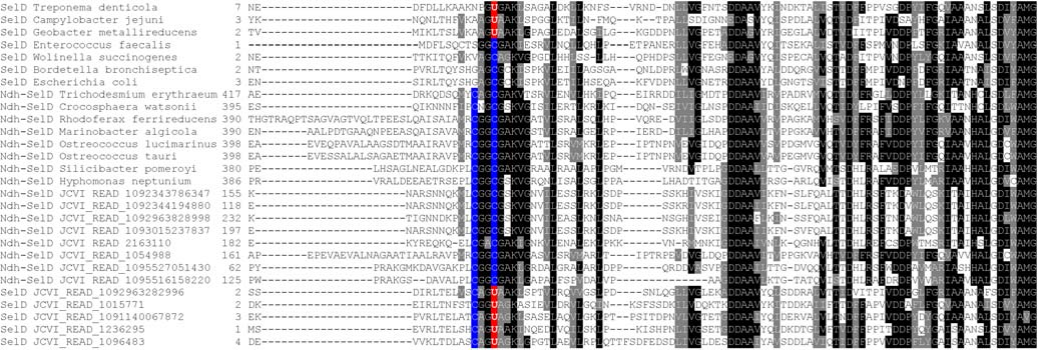
Multiple alignment of SelD. Conserved residues are highlighted. Predicted Sec (U) in selenoproteins and the corresponding Cys (C) residues in homologs are shown in red and blue background, respectively. Note that all Ndh-SelD fusion proteins contain a CxxC motif.

Previously, we detected two fusions of SelD: (i) NADH dehydrogenase (COG1252, Ndh, FAD-containing subunit) fusion [32] and (ii) Cys sulfinate desulfinase (COG1104, NifS) fusion (unpublished data). The Ndh-SelD fusion proteins were detected in several bacteria most of which were aquatic organisms. Such fusions were also observed in several lower eukaryotes, such as in *Ostreococcus*. In all detected fusion sequences, a conserved CxxC motif was present in the predicted active site of the SelD domain. However, this motif is very rare (<5%) in single-domain SelD proteins. The NifS-SelD fusion was only detected in *Geobacter sp. FRC-32* (an anaerobic, iron- and uranium-reducing deltaproteobacterium), and a CxxU motif was present in the active site of the SelD domain. Functions of the two fusion SelDs are not fully clear, but are expected to be involved in selenophosphate synthesis. In the GOS dataset, we detected hundreds of Ndh-SelD fusion proteins (all containing the CxxC motif), which accounted for approximately 40% of all detected Cys-containing SelDs. In contrast, no NifS-SelD fusion was detected. Interestingly, we found that ∼5.6% of single-domain selenoprotein SelDs contained a CxxU motif. Figure 12 shows a multiple alignment of Ndh-SelD fusion proteins and other Sec/Cys-containing SelDs in both sequenced organisms and GOS samples.

We also found several sequence reads containing two neighboring selenoprotein genes, including ten Prx/SelW sequences, one Prx/Prx-like 2 and one Prx-like 1/AhpD-like 2 sequences. Phylogenetic analysis showed that these Prx and SelW sequences were clustered in a small phylogenetic group, suggesting that they come from closely related organisms. Further analyses are needed to examine a possible functional link between these selenoproteins.

### Occurrence of the Selenouridine Utilization Trait in GOS Samples

In some prokaryotes, Se (in the form of selenophosphate) is also used for biosynthesis of a modified tRNA nucleoside, 5-methylaminomethyl-2-selenouridine (mnm^5^Se^2^U), which is located in the wobble position of the anticodons of tRNA^Lys^, tRNA^Glu^, and tRNA1^Gln^ [50]–[52]. The proposed function of mnm^5^Se^2^U involves codon-anticodon interactions that help base pair discrimination at the wobble position and/or translation efficiency [52],[53]. A 2-selenouridine synthase (YbbB) is necessary to replace a sulfur atom in 2-thiouridine in these tRNAs with selenium [54]. Here, we investigated the occurrence of YbbB to assess the selenouridine utilization trait in the GOS samples.

**Figure 13:**
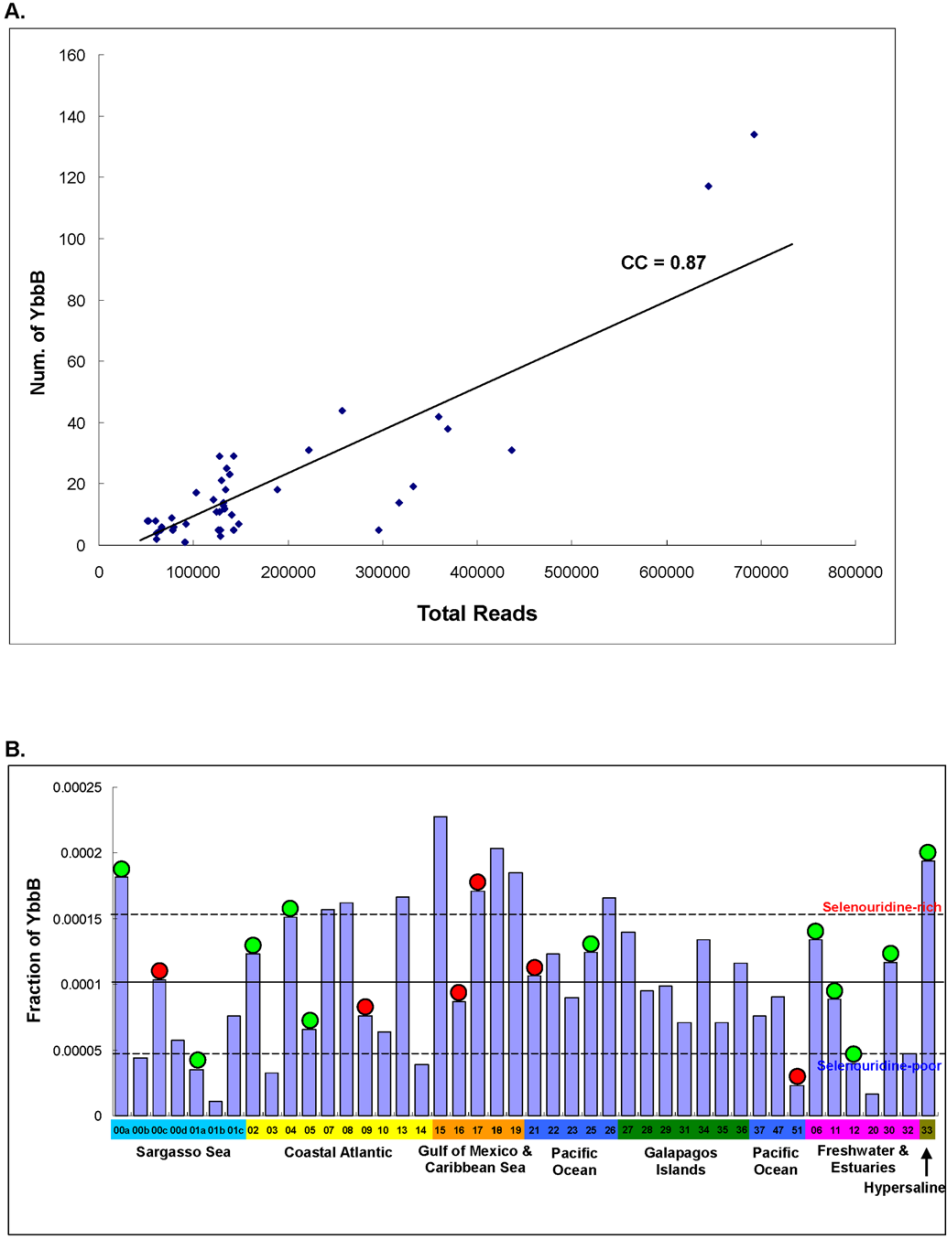
Distribution of selenouridine utilization trait in GOS samples. A. Distribution of YbbB in each GOS sample; B. Normalized occurrence of YbbB sequences. Colors represent different types of environments. Boundary lines for selenouridine-rich or selenouridine-poor samples are shown. Red circle, selenoprotein-rich sample; Green circle, selenoprotein-poor sample.

A total of 865 YbbB genes were identified in GOS sequences. Occurrence of YbbB in individual samples is shown in Figure 13A. In most GOS samples, the number of reads containing YbbB gene was proportional to the sample size (CC is 0.87). However, several samples appeared to have a significantly different distribution of YbbB. Similarly to selenoprotein classification of GOS samples, we clustered them into selenouridine-rich and selenouridine-poor. Previously, we have suggested a relatively independent relationship between Sec and selenouridine utilization [32]. In the current study, we examined correspondence between selenoprotein-rich/poor samples and selenouridine-rich/poor samples (Figure 13B). Two selenoprotein-poor samples (GS00a and GS33) were selenouridine-rich, whereas one selenoprotein-rich sample (GS51) appeared to be a selenouridine-poor sample, implying no strong relationship of the two Se utilization traits in GOS samples. Also, no significant difference was observed for the occurrence of the selenouridine utilization trait in other selenoprotein-rich/poor samples, further suggesting a relatively independent relationship between them. Considering that Se supply in the sea water should be equal to co-occurring Sec-utilizing and selenouridine-utilizing organisms, substantial microbial taxonomic diversity might explain differences in Se utilization in different areas of the sea. No clear relationship was also found between selenouridine utilization and habitat types or geographic location. Except for GS01a (a sample collected with a large filter), GS12 (from the estuary close to Chesapeake Bay, MD) was the only sample in which both Se utilization traits were limited. We also found high utilization of both traits in GS17 (Caribbean Sea, Yucatan Channel).

## Discussion

In recent years, a number of metagenomic sequencing projects were carried out that enabled researchers to identify genes in both abundant and non-abundant microbes in a particular environment, providing a powerful tool to examine community organization and metabolism in natural microbial communities [30], [55]–[57]. Similarly, identification of selenoprotein genes in these datasets may help in understanding the role of Se in microbial populations. In this study, we have used shotgun data from a recent GOS expedition [27]–[29] to characterize the distribution and composition of the selenoproteome in this largest to date marine metagenomic dataset. Our results highlight importance of Se utilization within marine microbial communities and provide a comprehensive analysis of Se-dependent proteins which are utilized by marine microorganisms.

The GOS project produced a total of 7.7 million random sequence reads from the North Atlantic Ocean, the Panama Canal, and East and central Pacific Ocean gyre. In order to identify all selenoproteins in the GOS dataset we employed a procedure that analyzed Sec/Cys pairs in homologous sequences. A total of 3,506 sequences which belonged to 51 previously described prokaryotic selenoprotein families, and 102 sequences that corresponded to 7 new selenoprotein families were identified. Compared to smaller scale non-aquatic metagenomic projects, such as whale fall community and Waseca County farm soil metagenome [56] and human distal gut microbiome [57], the GOS project produced hundreds of times more selenoproteins. Our current study generated by far the largest selenoproteome reported to date.

If selenoproteins and their Cys-containing homologs are randomly used in marine microbes, the number of selenoproteins would be expected to be proportional to the number of sequence reads in GOS samples. However, whereas the correlation was good for Cys homologs, it was weak for selenoproteins. We normalized the occurrence of selenoproteins in each sample and found that all selenoprotein-rich samples originated from the sea water and almost all from the tropical sea areas. In contrast, half of the selenoprotein-poor samples were obtained from nonmarine aquatic environments (including fresh and hypersaline water), and half of the marine selenoprotein-poor samples came from temperate sea areas. Thus, our data suggest that water salinity and temperature may influence Sec utilization. However, the fact that the occurrence of selenoproteins in some samples collected from sites with similar temperature and salinity was somewhat different suggests that additional factors may also affect Sec utilization. Moreover, other features of GOS samples (e.g., water depth, fraction filter and light intensity) may also result in bias when comparing the samples.

Among 51 previously characterized selenoprotein families, most were homologs of known thiol oxidoreductases or possessed Trx-like fold, consistent with the idea of redox function for selenoproteins in marine microorganisms. Twenty selenoprotein families, including SelW-like, SelD, Trx-like 1 and UGSC-containing proteins, were found to be the major selenoprotein families in GOS samples and represented approximately 95% of all detected selenoprotein sequences. Except for SelD, FdhA and UshA-like (COG0737, UshA, 5′-nucleotidase/2′,3′-cyclic phosphodiesterase and related esterases), all of these families contained conserved Cys-based redox motifs which are involved in a variety of redox functions. Comparison of the distributions of these major selenoprotein families in marine and nonmarine environments showed that a small number of selenoproteins exhibited significantly different occurrence in the two types of habitat. For example, SelW-like, DsbA 1, Prx-like 2, Prx-like 3 and Trx-like 3 were much more abundant in marine samples whereas UGSC-containing, AhpD-like 2 and Prx-like (UGC-containing) proteins were more abundant in nonmarine samples. Therefore, salinity and other factors affected the use of Sec, but this influence is not necessarily unidirectional and depends on specific selenoproteins affected.

Seven new selenoprotein families were identified. Except for hypothetical protein GOS_C, which was represented by 74 selenoprotein sequences in the GOS dataset, occurrence of other new selenoprotein families was limited. Among these new families, FTR is a well-characterized enzyme involved in disulfide reduction in Trx. However, previous studies could not detect any Sec-containing form for this enzyme. In addition, several Sec-containing sequences were predicted for a trypsin-like family, suggesting a potential redox function for a particular Cys residue in this well-known serine protease family. Although functions of other new families are unclear, the fact that a CxxU motif was present in both FmdB putative regulatory protein family and putative secreted serine protease MucD, and that a UxxC motif was present in a hypothetical protein GOS_C, implied a thiol-related redox function.

It has been reported that a small fraction (less than 3%) of reads in the GOS dataset is of eukaryotic origin (e.g., small-sized green algae). We did detect several eukaryotic selenoproteins, including a new selenoprotein family, GILT. Homologs of this protein family were only detected in eukaryotes. A eukaryotic SECIS element was detected in the 3′-UTR in one selenoprotein sequence. Although eukaryotic organisms containing the Sec-containing GILT are not known, future studies will likely identify such organisms.

Domain fusions could help identify functionally-related proteins. We identified several new fusion events involving selenoproteins. Compared to their more common forms present in most organisms, these selenoproteins contained additional upstream or downstream domains fused into a single protein chain. Fusion events were observed for a variety of Trx-fold-containing selenoproteins, including Prx, Prx-like 2, Prx-like 3 and UGSC-containing protein. Function of most of these fused domains is not clear; however, single or multiple conserved Cys residues were present in these domains, suggesting a potential redox function of these residues. In addition, almost half of the Cys-containing SelDs detected in the current GOS dataset were Ndh-SelD fusion proteins, all of which contained a conserved CxxC motif in the active sites. The abundance of Ndh-SelD fusion proteins in GOS samples suggests that this fusion SelD plays an important role in selenophosphate biosynthesis in marine/aquatic organisms.

Given that Se is also utilized for biosynthesis of selenouridine in bacteria, distribution of the selenouridine trait was assessed by analyzing occurrence of YbbB in GOS samples. We identified selenouridine-rich and selenouridine-poor samples, which were not the same as Sec-rich/poor samples, suggesting that the two Se utilization traits are functionally independent (but of course both depend on supply of Se). This observation is consistent with the previous hypothesis that Sec and selenouridine utilization traits are relatively independent even though both traits require SelD for selenophosphate biosynthesis [32]. In addition, no strong relationship was found between selenouridine utilization and habitat types (marine or nonmarine) or geographic location. Although both Se traits require Se supply or thus could influence evolution of each other, additional factors appear to play more important roles in the evolution and utilization of individual Se utilization traits.

In this study, we report a comprehensive analysis of Sec utilization in marine microbial samples of the GOS expedition by characterizing the GOS selenoproteome. This is the first time that the microbial selenoprotein population is described in a global biogeographical context. Our analysis yielded the largest selenoprotein dataset to date, provided a variety of new insights into Sec utilization and revealed environmental factors that influence Sec utilization in the marine microbial world.

## Materials and Methods

### GOS Sequence Resource

Shotgun reads of the GOS project were downloaded from the CAMERA (Community Cyberinfrastructure for Advanced Marine Microbial Ecology Research and Analysis) website at http://camera.calit2.net. This dataset contains a total of 7,709,422 genomic sequences derived from 57 samples (13 samples are not fully sequenced), which cover a wide range of distinct surface marine environments as well as a few nonmarine aquatic samples [29]. The genomic sequences combined had 8.148 billion nucleotides. In addition, we downloaded the non-redundant (NR) protein database from the NCBI ftp server. It contained a total of 4,644,764 protein sequences. BLAST [58] was also obtained from the NCBI.

### High Throughput Computing Resource

Previously, we developed and employed a set of programs for automated selenoprotein searches [24]–[26]. However, since this approach is based on an exhaustive search of all possible Cys/Sec pairs for each Cys-containing sequence in the NR database, the computation procedure can become very expensive when the target sequence dataset is very large, as is the case in the GOS database. Therefore, we utilized an Open Science Grid (OSG) management system which is dedicated to supporting scientific research through the use of advanced networking technology and high performance computing [59]. We employed Condor-G software [60], a powerful and full-featured task broker, to manage such a high throughput computing project on large collections of distributively owned computing resources. In addition, we used the Prairiefire, a 128-node, 256-processor Beowulf cluster supercomputer at the Research Computing Facility of the University of Nebraska – Lincoln.

### Search Procedure

We used a strategy which we had successfully used in selenoprotein searches in other metagenomic datasets: Sargasso Sea and symbiotic microbial consortium of the marine oligochaete *Olavius algarvensis* [24]–[26]. Briefly, each Cys-containing sequence in the NR protein database was searched against the GOS dataset for top 1000 homologs using TBLASTN with E-value below 10 (this step is the most time-consuming and was performed completely on the OSG system). Cys/TGA pairs were then selected and a minimum open reading frame (ORF) for each TGA-containing nucleotide sequence (TGA was translated to Sec, U) was obtained. After that, BLASTX and RPS-BLAST programs were used to analyze the conservation of TGA-flanking regions in all six reading frames as well as the presence of conserved domains. Remaining sequences were clustered based on sequence similarity and location of predicted Sec using BL2SEQ with an E-value below 10^−4^. All clusters were further searched against NCBI NR protein and microbial genomic databases to identify conserved Cys-containing homologs. Sequences in the remaining clusters were manually analyzed for occurrence of bacterial SECIS elements using bSECISearch program [21], and were classified into known selenoproteins and candidate selenoproteins (i.e., clusters having at least two Sec-containing sequences). In addition, an independent BLAST homology search for selected Sec-containing representatives of all previously identified prokaryotic selenoprotein families was performed. Finally, distinct representatives of all identified selenoprotein sequences were used to iteratively search against the GOS dataset for identification of additional distant Sec-containing homologs.

### Multiple Sequence Alignment and Phylogenetic Analysis

We used CLUSTALW [61] with default parameters for multiple sequence alignments. Phylogeny was analyzed by PHYLIP programs [62]. Neighbor-joining (NJ) trees were obtained with NEIGHBOR and the most parsimonious trees were determined with PROTPARS. Robustness of these phylogenies was evaluated by two additional algorithms: maximum likelihood (ML) analysis with PHYML [63] and Bayesian estimation of phylogeny with MrBayes [64].

## Supporting Information

Figure S1 Additional fusion selenoproteins. A. UGSC/Unknown_1 fusion; B. Prx-like 2/Distant Secretin_N fusion; C. Unknown_2/Prx-like 2 fusion; D. Prx-like 3/Unknown_3 fusion. Only the alignments of fused domains are shown. The conserved Cys residues in different domains are highlighted in pink background.
(0.11 MB PDF)

Table S1 Distribution of selenoproteins in individual samples.
(0.04 MB XLS)

Dataset S1 Sequences of homologs of known selenoproteins.
(0.60 MB TXT)

Dataset S2 Sequences of new selenoproteins.
(0.02 MB TXT)
