# Supplementary material for: Trends in Selenium Utilization in Marine Microbial World Revealed through the Analysis of the Global Ocean Sampling (GOS) Project
Source: PLoS Genet. 2008 Jun 13;4(6):e1000095. doi: 10.1371/journal.pgen.1000095 (PMC2398784; doi:10.1371/journal.pgen.1000095)
Supplement: Figure S1 — Additional fusion selenoproteins. A. UGSC/Unknown_1 fusion; B. Prx-like 2/Distant Secretin_N fusion; C. Unknown_2/Prx-like 2 fusion; D. Prx-like 3/Unknown_3 fusion. Only the alignments of fused domains are shown. The conserved Cys residues in different domains are highlighted in pink background. (0.11 MB PDF) [file pgen.1000095.s001.pdf]

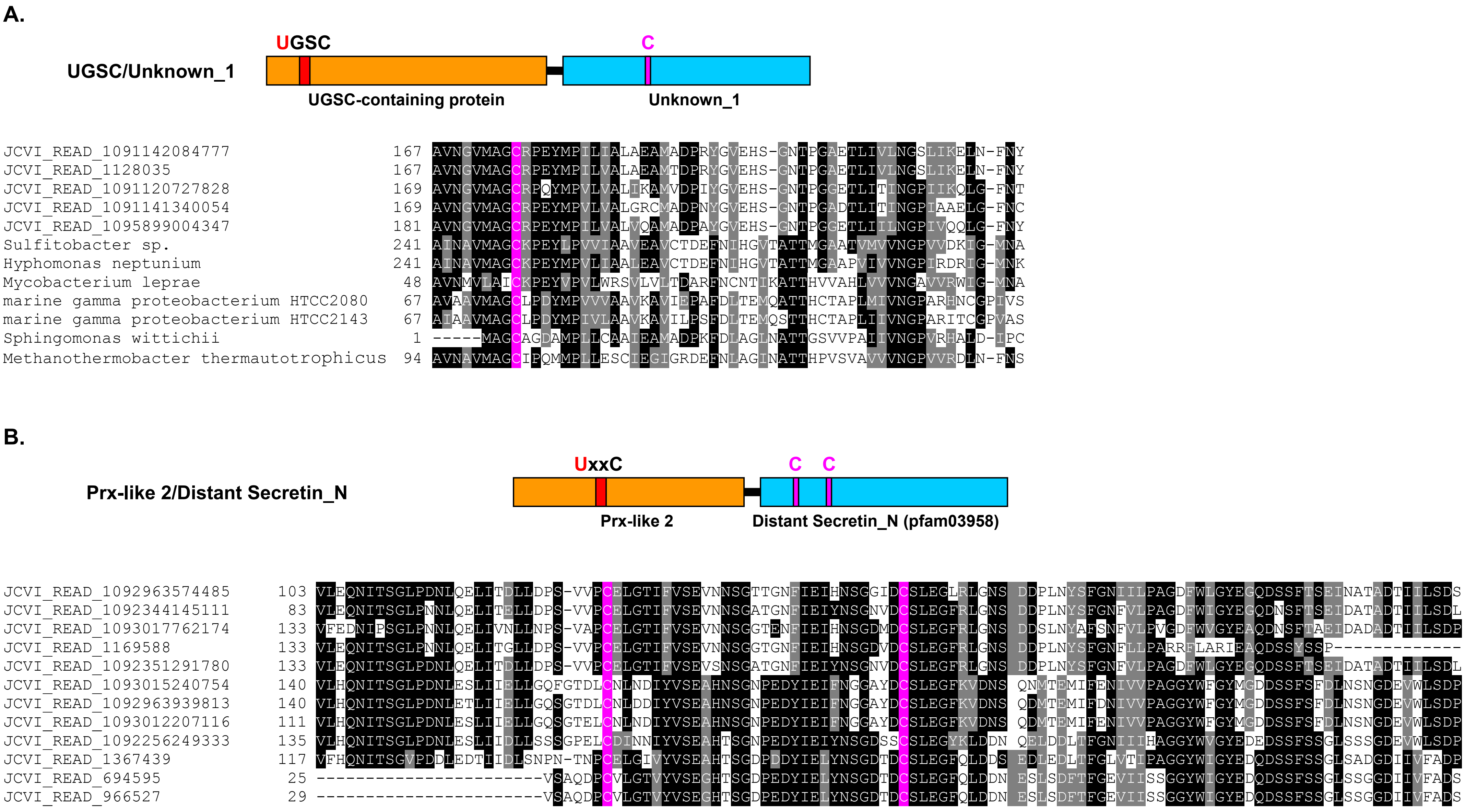

A.
UGSC
C
UGSC/Unknown_1
UGSC-containing protein
Unknown_1
JCVI_READ_1091142084777 167 AVNGVMAGCRPEYMPILIALAEAMADPRYGVEHS-GNTPGAETLIVLNGSLIKELN-FNY
JCVI_READ_1128035 167 AVNGVMAGCRPEYMPILVALAEAMTDPRYGVEHS-GNTPGAETLIVLNGSLIKELN-FNY
JCVI_READ_1091120727828 169 AVNGVMAGCRPQYMPVLVALIKAMVDPIYGVEHS-GNTPGGETLITINGPIIKQLG-FNT
JCVI_READ_1091141340054 169 AVNGVMAGCRPEYMPVLVALGRCMADPNYGVEHS-GNTPGADTLITINGPIAAELG-FNC
JCVI_READ_1095899004347 181 AVNGVMAGCRPEYMPILVALVQAMADPAYGVEHS-GNTPGGETLIILNGPIVQQLG-FNY
Sulfitobacter sp. 241 AINAVMAGCKPEYLPVVIAAVEAVCTDEFNIHGVTATTMGAATVMVVNGPVVDKIG-MNA
Hyphomonas neptunium 241 AINAVMAGCKPEYMPVLIAALEAVCTDEFNIHGVTATTMGAAPVIVVNGPIRDRIG-MNK
Mycobacterium leprae 48 AVNMVLAICKPEYVPVLWRSVLVLTDARFNCNTIKATTHVVAHLVVVNGAVVRWIG-MNA
marine gamma proteobacterium HTCC2080 67 AVAAVMAGCLPDYMPVVVAAVKAVIEPAFDLTEMQATTHCTAPLMIVNGPARHNCGPIVS
marine gamma proteobacterium HTCC2143 67 AIAAVMAGCLPDYMPIVLAAVKAVILPSFDLTEMQSTTHCTAPLIIVNGPARITCGPVAS
Sphingomonas wittichii 1 -----MAGCAGDAMPLLCAAIEAMADPKFDLAGLNATTGSVVPAIIVNGPVRHALD-IPC
Methanothermobacter thermautotrophicus 94 AVNAVMAGCIPQMMPLLESCIEGIGRDEFNLAGINATTHPVSVAVVVNGPVVRDLN-FNS
B.
UxxC
C C
Prx-like 2/Distant Secretin_N
Prx-like 2
Distant Secretin_N (pfam03958)
JCVI_READ_1092963574485 103 VLEQNITSGLPDNLQELITDLLDPS-VVPCELGTIFVSEVNNSGTTGNFIEIHNSGGIDCSLEGLRLGNS DDPLNYSFGNIILPAGDFWLGYEGQDSSFTSEINATADTIILSDS
JCVI_READ_1092344145111 83 VLEQNITSGLPNNLQELITELLDPS-VVPCELGTIFVSEVNNSGATGNFIEIYNSGNVDCSLEGFRLGNS DDPLNYSFGNFVLPAGDFWIGYEGQDNSFTSEIDATADTIILSDL
JCVI_READ_1093017762174 133 VFEDNIPSGLPNNLQELIVNLLNPS-VAPCELGTIFVSEVNNSGGTENFIEIHNSGDMDCSLEGFRLGNS DDSLNYAFSNFVLPVGDFWVGYEAQDNSFTAEIDADADTIILSDP
JCVI_READ_1169588 133 VLEQNITSGLPNNLQELITGLLDPS-VVPCELGTIFVSEVNNSGGTGNFIEIHNSGDVDCSLEGFRLGNS DDPLNYSFGNFLLPARRFLARIEAQDSSYSSP-------------
JCVI_READ_1092351291780 133 VLEQNITSGLPDNLQELITDLLDPS-VVPCELGTIFVSEVSNSGATGNFIEIYNSGNVDCSLEGFRLGNS DDPLNYSFGNFVLPAGDFWLGYEGQDSSFTSEIDATADTIILSDL
JCVI_READ_1093015240754 140 VLHQNITSGLPDNLESLIIELLGQFGTDLCNLNDIYVSEAHNSGNPEDYIEIFNGGAYDCSLEGFKVDNS QNMTEMIFENIVVPAGGYWFGYMGDDSSFSFDLNSNGDEVWLSDP
JCVI_READ_1092963939813 140 VLHQNITSGLPDNLETLIIELLGQSGTDLCNLDDIYVSEAHNSGNPEDYIEIFNGGAYDCSLEGFKVDNS QDMTEMIFDNIVVPAGGYWFGYMGDDSSFSFDLNSNGDEVWLSDP
JCVI_READ_1093012207116 111 VLHQNITSGLPDNLESLIIELLGQSGTELCNLNDIYVSEAHNSGNPEDYIEIFNGGAYDCSLEGFKVDNS QDMTEMIFENIVVPAGGYWFGYMGDDSSFSFDLNSNGDEVWLSDP
JCVI_READ_1092256249333 135 VLHQNITSGLPDNLESLIIDLLSSSGPELCDINNIYVSEAHTSGNPEDYIEIYNSGDSSCSLEGYKLDDN QELDDLTFGNIIIHAGGYWVGYEDEDSSFSSGLSSSGDEVWLSDP
JCVI_READ_1367439 117 VFHQNITSGVPDDLEDTIIDLSNPN-TNPCELGIVYVSEAHTSGDPDDYIELYNSGDTDCSLEGFQLDDS EDLEDLTFGLVTIPAGGYWIGYEDQDSSFSSGLSADGDIIVFADP
JCVI_READ_694595 25 -----------------------VSAQDPCVLGTVYVSEGHTSGDPEDYIELYNSGDTDCSLEGFQLDDN ESLSDFTFGEVIISSGGYWIGYEDQDSSFSSGLSSGGDIIVFADS
JCVI_READ_966527 29 -----------------------VSAQDPCVLGTVYVSEGHTSGDPEDYIELYNSGDTDCSLEGFQLDDN ESLSDFTFGEVIISSGGYWIGYEDQDSSFSSGLSSGGDIIVFADS

C.

Unknown_2/Prx-like 2

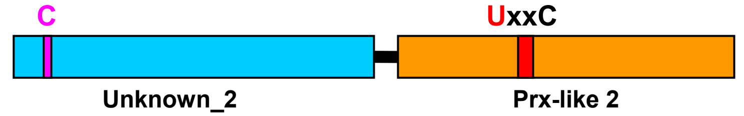


```
JCVI_READ_1091141392542 1 ------------------------------------------MASLLETCAKVNAESYYWIVVLPFEG-T NAKWVYSTENTFEYSTSTDGGYSWSQYNSGFAGTSYLTAEQIYIP--PF
JCVI_READ_109112090526  1 ------------------------------------------MASLLETCAKVNAESYYWIVVLPFEG-T NAKWVYSTENTFEYSTSTDGGYSWSQYNSGFAGTSYLTAEQIYIP--PF
JCVI_READ_1091140728852 1 ------------------------------------NLTGYNLIVTTDLCIYLDSNTPYWLRIDAADTST QAIWSYSAGSLYTYSENTN--QSGWLNTIGATGASGIFAEQIYDR--PY
JCVI_READ_1093016277959 1 -MKSDINSTITVKLFSDNFNSPGDLIYEWPLNLSASDFFAEIYTPTVSFCINLTSNSYYWLSVETPDSVA NISWQHSILN--SYYSTSDNGIDWSESIYGPVGATSIRGEQIFTFSQGY
JCVI_READ_1624171       1 MSKESDFSSVLISVHEDSDNSPGQEIGNWVLSLS-TNTPREYTIYTLDECITFNANQNYWLSVKANDETS SARWIYSPSDSYNYSISTDGQETWTSS-LGFAGSTKIYAEIFYAP--DP
```

D.

Prx-like 3/Unknown_3

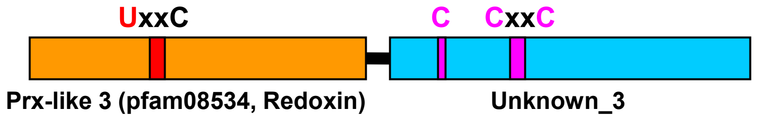


```
JCVI_READ_1095403593256 140 SYVPRDQDGDGVMDSNDNCVAVSNYHQDDYDLDGSGDACDPCNNLDIYVAGNIDGTQNSINSEAIIDIFDVLSLTDIVLKGIDY-----GCGYEISDIREDGNLNVLDIVALVQIVL
JCVI_READ_1095901429861 179 SYVPRDQDGDGIMDNNDNCIEISNYHQDDYDLDGSGDACDPCNNLDIFITGNIDGTLSPFDGGATIDIFDILSLTDIVLEGTED-----GCGHEISDIRQDGDVNVLDIITLVQLIL
JCVI_READ_1304752       158 ELMQEDADGDGLILD-DNCPEDYNPSQFDDDEDGIGDECDNC-NNNIFIPGNLDGTL-ELDGSLSLNVLDLLLLSDIVNEEPED-----GCAITATDITGDGVTNIIDVYAFASMIL
JCVI_READ_1351145       167 ENM--DADNDGVYNSMDNCPDIPNPYQEDEDGDGIGDACDVC-NSVVFYNGNLNGDE-------SINIIDVIMLIDVILGIDTN-----VCAYESSDINNDDIVNVLDVINLIQEIL
JCVI_READ_1092256010056 164 NTIIPDLDNDGVLDNIDNCVDIYNPEQSDTDLDNIGDECDECDNLNIFVEENIYGEIDSLN-NFTIDIFDLLTLVDIILSNDIE-----NCGFYIGDLTNDGLVNVFDVIALSQVIL
JCVI_READ_1093012211543 164 NTIIPDFDNDGVLDNVDNCVDIYNPEQIDTDLDNIGNECDNCDNLNIFVDENIMRLPTV----------------------------------------------------------
JCVI_READ_1091140693769 164 STIIQDSDSDNILDNIDNCINIYNPEQNDYDFDHIGDECDSCDNLNIFINGNIYGDIDNEN-NYSIDIFDLLTLLDIIIYDYDN-----LCGYQIADITGDGQQNILDAISLIQIIM
JCVI_READ_1093015751885 109 NTISGDYDGDGILDDVDNCIDVHNSLQNDNDLDGIGDACDSCDNLFVYVDGNIYGEVDYQ S-NYDIDIFDLITLMDIIANDNTN-----NCGYEIGDITNDGNVNIIDAIALLQRI
JCVI_READ_1095898056526 160 EDLPSDLDEDGIDTDEDNCPENFNPLQSDIDEDGSGDACDICDNVNVFTTGNVNGDLDENN-FPIINFFDVVALLDHFQLNESELLPISECREQAGNINGDNNVNIIDVVNLVNMVL
JCVI_READ_1095898023484 160 EDLPSDLDEDGIDTDEDNCPENFNPLQSDIDEDGSGDACDICDNVNVFTTGNVNGDLDENN-FPIINFFDVVALLDHFQLNESELLPISECREQAGNINGDNNVNIIDVVNLVNMGV
JCVI_READ_1092963742291 161 EDLPSDLDEDGFDFDEDNCPETYNPAQSDIDEDGSGDACDICDNANVFIVGNVNGDLDDNN-NPIIDFFDVVSLLDHLQTDEANETPISECRQQAANINYDNNVNIIDVVNLVNMIL
JCVI_READ_1095368019320 161 EDLPSDLDEDGFDFDEDNCPETYNPAQSDIDEDGSGDACDICDNANVFIVGNVNGDLDDNN-NPIIDFFDVVSLLDHLQTDEANETPISECRQQAANSLHPRT--------------
JCVI_READ_1091143199283 159 EDLPSDLDEDGFDVDIDNCPEIYNPTQADIDEDGKGDACDICDNANIYVVGNVNGDLDESG-SPKINFFDVVALLDHLQLDDSEEIPISECRQQAGNINYDNNVNIIDVVNLVNMVL
JCVI_READ_1092255327168 148 EDLPSDFDEDGFDEDLDNCPENYNPTQADIDGDGLGDACDVCDNENVFVSGNVNGDIDLNN-EPIIDFFDVVALLDYLQLSQTTPQAITECQAQSGNINADNNVNIIDVVNLVNILL
JCVI_READ_1093017425796 132 EDLPSDFDEDGFDEDVDNCPENYNPTQADIDGDGYGDACDVCDNENVFITGNVNGDIDLNN-EPIIDFFDVVALLDHLQLSQAAPQAISECEAQSGNINADNNVNIIDVVNLVNMLL
JCVI_READ_1091145068910 115 EDLPSDLDEDGVDTDEDNCPESFNPLQTDLDEDGSGDACDICDNANVFTTGNVNGDLDEGN-LPLINFF------------------------------------------------
JCVI_READ_1091141649070 114 ENLILDVDEDGVLDSDDNCIDIANPNQADIDFDGAGDACDPCDNQNVYTFGNINGTIDQEG-IAIVDIFDVMELVDILLSDDNE-----SCGSEIADMNSDGNQNVVDIIFLVQMLL
JCVI_READ_1092255434378  40 EDLPSDFDEDGFDEDLDNCPENYNPTQADIDGDGLGDACDVCDNENVFVSGNVNGDIDLNN-EPIIDFFDVVALLDYLQLSQTTPQAITECQAQSGNINADNNVNIIDVVNLVNILL
JCVI_READ_1092963182296  27 EDLPSDLDEDGFDVDIDNCPEIYNPTQSDIDEDGKGDACDICDNANVYVVGNVNGDLDESG-SPKINFFDVVALLDYLQLDDSEEIPISECRQQAGNINYDNNVNIIDVVNLVNMVL
JCVI_READ_916441          1 MGVNQDIDEDGIIAEEDNCPETYNPAQNDLDIDGYGDACDICDNANVFVMGNVNGDLSITN-EPKIDFFDVVSLIDHISVSDET--AMSECSAQAGNINGDNNVNIIDVVNLVNMLL
```
